# Supplementary material for: Single-cell RNA-seq data have prevalent blood contamination but can be rescued by Originator, a computational tool separating single-cell RNA-seq by genetic and contextual information
Source: Genome Biol. 2025 Mar 11;26:52. doi: 10.1186/s13059-025-03495-9 (PMC11895284; doi:10.1186/s13059-025-03495-9)
Supplement: Supplementary file 1 — Additional file 1: Tables S1–S12. [file 13059_2025_3495_MOESM1_ESM.docx]

**Supplementary Tables**

**Table S1: Comparison between Freemuxlet and scSplit in assigning cells according to their genetic origins.** Table S1a-b show the performance of Freemuxlet and scSplit in (a) assigning trophoblast cells to the fetal origin in the placenta and (b) assigning cells according to genetic origin on mixed PBMC data from two clear cell renal cell carcinoma patients.

Table S1a

| **Placenta tissue samples** | **Tools** | **Correct trophoblast assignment to the fetal origin (%)** |
| --- | --- | --- |
| Placenta sample 1 | Freemuxlet | 100 |
|  | scSplit | 52.24 |
| Placenta sample 2 | Freemuxlet | 100 |
|  | scSplit | 61.92 |

Table S1b

| **Tools** | **F1 score** |
| --- | --- |
| Freemuxlet | 0.97 |
| scSplit | 0.73 |

**Table S2: Average performance of 5 executions of blood and expected tissue-resident immune cell identification on the artificially-mixed blood-tissue resident data measured by four metrics including area under receiver operating characteristic curve (AUC), area under precision-recall curve (AUCPR), F1 score, and Matthews correlation coefficient (MCC).**

| **Cell type** | **AUC** | **AUCPR** | **F1 score** | **MCC** |
| --- | --- | --- | --- | --- |
| **T-cell** | 0.960  (SD = 0.001033) | 0.970  (SD = 0.000654) | 0.980  (SD = 0.000448) | 0.940  (SD = 0.001345) |
| **B-cell** | 0.998  (SD = 0) | 0.996  (SD = 0) | 0.997  (SD = 0) | 0.988  (SD = 0) |
| **monocyte** | 0.939  (SD = 0.007768) | 0.913  (SD = 0.007877) | 0.939  (SD = 0.005704) | 0.830  (SD = 0.015872) |

| **Overall performance** | | | |
| --- | --- | --- | --- |
| **AUC** | **AUCPR** | **F1 score** | **MCC** |
| 0.958  (SD = 0.002483) | 0.957  (SD = 0.002472) | 0.971  (SD = 0.001714) | 0.909  (SD = 0.005270) |

**Table S3: Top significant DE genes comparing expected tissue-resident and blood T-cells in PDAC tumor tissue, as identified by Originator.** log2 fold change (avg_log2FC) is the averaged expression in expected tissue-resident compared to blood T-cells.

|  | **p_val** | **avg_log2FC** | **pct.1** | **pct.2** | **p_val_adj** |
| --- | --- | --- | --- | --- | --- |
| CCL4 | 2.12E-36 | 1.573474 | 0.277 | 0.118 | 6.95E-32 |
| GZMK | 2.37E-60 | 1.333029 | 0.377 | 0.144 | 7.76E-56 |
| CCL5 | 2.94E-75 | 1.284637 | 0.507 | 0.222 | 9.62E-71 |
| PARP8 | 3.59E-60 | 1.284428 | 0.604 | 0.446 | 1.17E-55 |
| AOAH | 2.10E-14 | 1.034958 | 0.201 | 0.116 | 6.87E-10 |
| ZEB2 | 1.78E-21 | 1.004127 | 0.335 | 0.222 | 5.82E-17 |
| CBLB | 6.15E-22 | 0.952122 | 0.496 | 0.436 | 2.01E-17 |
| FOSB | 1.16E-21 | 0.868436 | 0.477 | 0.398 | 3.81E-17 |
| FKBP5 | 4.16E-14 | 0.808336 | 0.387 | 0.322 | 1.36E-09 |
| NKG7 | 6.24E-23 | 0.802629 | 0.18 | 0.071 | 2.04E-18 |
| GZMA | 3.41E-21 | 0.802536 | 0.311 | 0.193 | 1.12E-16 |
| ID2 | 2.56E-09 | 0.792395 | 0.291 | 0.237 | 8.38E-05 |
| METRNL | 3.48E-13 | 0.788526 | 0.234 | 0.155 | 1.14E-08 |
| ARHGAP26 | 8.32E-13 | 0.752598 | 0.353 | 0.288 | 2.72E-08 |
| CST7 | 1.02E-09 | 0.752057 | 0.218 | 0.156 | 3.34E-05 |
| TSC22D3 | 4.26E-20 | 0.734774 | 0.596 | 0.58 | 1.40E-15 |
| MT-ND1 | 7.77E-104 | 0.730171 | 0.98 | 0.989 | 2.54E-99 |
| NFE2L3 | 1.12E-11 | 0.72918 | 0.14 | 0.074 | 3.65E-07 |
| ZSWIM4 | 8.14E-09 | 0.711702 | 0.127 | 0.072 | 0.000266 |
| MT-CO1 | 2.42E-131 | 0.708822 | 0.995 | 0.997 | 7.93E-127 |
| CD8A | 8.88E-15 | 0.708242 | 0.109 | 0.041 | 2.91E-10 |
| AUTS2 | 1.51E-10 | 0.690459 | 0.247 | 0.175 | 4.96E-06 |
| PLCB1 | 1.24E-11 | 0.68456 | 0.279 | 0.199 | 4.04E-07 |
| MT-CO3 | 6.79E-116 | 0.680299 | 0.992 | 0.997 | 2.22E-111 |
| SLC7A5 | 1.18E-08 | 0.659229 | 0.297 | 0.251 | 0.000386 |
| SLC35F1 | 8.71E-09 | 0.640933 | 0.245 | 0.187 | 0.000285 |
| MT-ATP6 | 1.13E-116 | 0.637472 | 0.998 | 1 | 3.71E-112 |
| MT-ND5 | 1.34E-34 | 0.621219 | 0.781 | 0.814 | 4.39E-30 |
| RALGAPA1 | 5.56E-10 | 0.60728 | 0.54 | 0.564 | 1.82E-05 |
| MT-ND2 | 1.46E-55 | 0.597805 | 0.961 | 0.976 | 4.77E-51 |
| MT-CYB | 1.28E-68 | 0.574227 | 0.989 | 0.992 | 4.20E-64 |
| MT-CO2 | 1.70E-95 | 0.560024 | 0.995 | 0.997 | 5.57E-91 |
| CTSW | 1.02E-08 | 0.503019 | 0.108 | 0.057 | 0.000333 |
| MT-ND4 | 5.17E-57 | 0.494535 | 0.977 | 0.994 | 1.69E-52 |
| MT-ND3 | 1.43E-47 | 0.462944 | 0.978 | 0.99 | 4.68E-43 |
| SIK3 | 1.29E-07 | 0.436137 | 0.695 | 0.792 | 0.004238 |
| FOS | 2.23E-07 | 0.309387 | 0.554 | 0.517 | 0.007291 |
| SUMO2 | 2.00E-24 | -0.25005 | 0.238 | 0.46 | 6.53E-20 |
| SERF2 | 4.47E-20 | -0.25007 | 0.482 | 0.737 | 1.46E-15 |
| RPS19 | 1.02E-21 | -0.25049 | 0.928 | 0.976 | 3.35E-17 |
| BTG2 | 1.20E-20 | -0.25189 | 0.305 | 0.522 | 3.93E-16 |
| ERO1L | 5.18E-20 | -0.25264 | 0.065 | 0.169 | 1.70E-15 |
| CCM2 | 5.45E-22 | -0.2527 | 0.095 | 0.224 | 1.78E-17 |
| OGDH | 4.05E-24 | -0.2536 | 0.189 | 0.382 | 1.33E-19 |
| TTC39C | 6.61E-26 | -0.25397 | 0.284 | 0.519 | 2.17E-21 |
| EIF3F | 2.11E-26 | -0.25411 | 0.179 | 0.379 | 6.90E-22 |
| CD69 | 1.55E-11 | -0.25552 | 0.343 | 0.523 | 5.07E-07 |
| COMMD6 | 1.46E-27 | -0.25558 | 0.279 | 0.528 | 4.78E-23 |
| RPL38 | 7.96E-19 | -0.25615 | 0.715 | 0.894 | 2.61E-14 |
| RPL27A | 6.41E-24 | -0.25647 | 0.86 | 0.967 | 2.10E-19 |
| KRT10 | 9.37E-23 | -0.25653 | 0.111 | 0.255 | 3.07E-18 |
| TRAF3 | 3.02E-26 | -0.25657 | 0.179 | 0.376 | 9.90E-22 |
| TET2 | 2.32E-20 | -0.25666 | 0.11 | 0.242 | 7.60E-16 |
| CTSB | 2.13E-22 | -0.25709 | 0.06 | 0.169 | 6.98E-18 |
| TNFSF8 | 1.36E-17 | -0.2571 | 0.072 | 0.172 | 4.44E-13 |
| RPL36A | 1.56E-22 | -0.25711 | 0.398 | 0.653 | 5.11E-18 |
| ZNF706 | 5.81E-24 | -0.25727 | 0.085 | 0.217 | 1.90E-19 |
| MORC3 | 4.50E-24 | -0.25917 | 0.106 | 0.25 | 1.47E-19 |
| H3F3A | 5.01E-22 | -0.2592 | 0.527 | 0.783 | 1.64E-17 |
| NR3C1 | 1.54E-16 | -0.25921 | 0.416 | 0.621 | 5.04E-12 |
| HMGN1 | 1.04E-24 | -0.25943 | 0.178 | 0.369 | 3.39E-20 |
| ZBTB24 | 1.57E-19 | -0.2596 | 0.03 | 0.107 | 5.13E-15 |
| ARID5A | 5.53E-22 | -0.26048 | 0.151 | 0.312 | 1.81E-17 |
| ABRACL | 4.56E-22 | -0.26115 | 0.119 | 0.265 | 1.49E-17 |
| TRAPPC1 | 3.08E-23 | -0.26133 | 0.057 | 0.167 | 1.01E-18 |
| RPL21 | 7.28E-21 | -0.26149 | 0.863 | 0.954 | 2.38E-16 |
| GIMAP7 | 9.18E-28 | -0.26247 | 0.158 | 0.348 | 3.01E-23 |
| ATP5G2 | 7.22E-28 | -0.26249 | 0.262 | 0.508 | 2.37E-23 |
| TMBIM6 | 2.95E-28 | -0.26293 | 0.228 | 0.46 | 9.65E-24 |
| LAMTOR4 | 4.77E-30 | -0.26369 | 0.121 | 0.3 | 1.56E-25 |
| CDKN1A | 7.86E-22 | -0.26407 | 0.111 | 0.249 | 2.57E-17 |
| EEF2 | 7.16E-25 | -0.26466 | 0.321 | 0.584 | 2.35E-20 |
| PPA1 | 7.44E-22 | -0.26517 | 0.085 | 0.208 | 2.44E-17 |
| TOMM20 | 7.48E-27 | -0.2653 | 0.198 | 0.41 | 2.45E-22 |
| FAM107B | 9.17E-18 | -0.26639 | 0.415 | 0.636 | 3.00E-13 |
| PPDPF | 6.51E-26 | -0.26727 | 0.105 | 0.256 | 2.13E-21 |
| SLAMF1 | 5.30E-19 | -0.2673 | 0.061 | 0.159 | 1.74E-14 |
| ATP6V1F | 6.91E-25 | -0.26837 | 0.067 | 0.189 | 2.26E-20 |
| TMEM219 | 2.89E-26 | -0.26839 | 0.058 | 0.179 | 9.46E-22 |
| CLIC1 | 1.05E-27 | -0.26883 | 0.211 | 0.429 | 3.45E-23 |
| CMTM8 | 1.24E-16 | -0.27032 | 0.054 | 0.138 | 4.05E-12 |
| STAM | 2.59E-22 | -0.27097 | 0.07 | 0.185 | 8.48E-18 |
| RPS23 | 5.93E-26 | -0.27148 | 0.858 | 0.949 | 1.94E-21 |
| SRP9 | 9.09E-23 | -0.27225 | 0.061 | 0.171 | 2.97E-18 |
| BTF3 | 4.18E-23 | -0.27333 | 0.403 | 0.666 | 1.37E-18 |
| CDKN1B | 6.06E-25 | -0.27377 | 0.178 | 0.365 | 1.98E-20 |
| IL4I1 | 3.52E-12 | -0.27403 | 0.05 | 0.116 | 1.15E-07 |
| RAC2 | 1.19E-26 | -0.27489 | 0.134 | 0.305 | 3.91E-22 |
| PPP1CB | 1.42E-22 | -0.27516 | 0.31 | 0.549 | 4.65E-18 |
| PAG1 | 3.47E-23 | -0.27572 | 0.241 | 0.447 | 1.14E-18 |
| NDFIP1 | 2.41E-27 | -0.27587 | 0.231 | 0.457 | 7.88E-23 |
| P2RY10 | 1.14E-21 | -0.27598 | 0.089 | 0.213 | 3.74E-17 |
| BRK1 | 2.48E-27 | -0.27626 | 0.09 | 0.236 | 8.12E-23 |
| BAZ1A | 1.65E-20 | -0.27982 | 0.271 | 0.475 | 5.42E-16 |
| RPL37 | 9.57E-24 | -0.28 | 0.807 | 0.942 | 3.13E-19 |
| HIF1A | 1.65E-30 | -0.2805 | 0.118 | 0.293 | 5.41E-26 |
| PRELID1 | 2.52E-28 | -0.28055 | 0.113 | 0.278 | 8.25E-24 |
| KRAS | 5.96E-25 | -0.28062 | 0.135 | 0.3 | 1.95E-20 |
| NAB1 | 1.09E-21 | -0.28068 | 0.04 | 0.13 | 3.56E-17 |
| UBE2D2 | 3.20E-29 | -0.2834 | 0.249 | 0.497 | 1.05E-24 |
| RPL8 | 6.47E-24 | -0.28401 | 0.758 | 0.917 | 2.12E-19 |
| RPL30 | 3.73E-30 | -0.28418 | 0.894 | 0.961 | 1.22E-25 |
| CYTH1 | 1.04E-23 | -0.28439 | 0.368 | 0.63 | 3.40E-19 |
| RHOG | 2.65E-25 | -0.28479 | 0.082 | 0.217 | 8.66E-21 |
| ATRAID | 2.45E-23 | -0.28488 | 0.043 | 0.142 | 8.03E-19 |
| RPL36 | 1.48E-27 | -0.28539 | 0.768 | 0.929 | 4.84E-23 |
| INPP4B | 1.06E-22 | -0.28597 | 0.28 | 0.493 | 3.49E-18 |
| EIF3G | 9.61E-27 | -0.28671 | 0.151 | 0.331 | 3.15E-22 |
| CAPG | 3.00E-22 | -0.2885 | 0.042 | 0.136 | 9.82E-18 |
| ADAM12 | 1.00E-15 | -0.28883 | 0.049 | 0.126 | 3.28E-11 |
| VAMP8 | 4.46E-26 | -0.28914 | 0.11 | 0.265 | 1.46E-21 |
| RPS29 | 6.22E-32 | -0.29315 | 0.898 | 0.971 | 2.04E-27 |
| CD3D | 2.23E-25 | -0.29348 | 0.458 | 0.742 | 7.30E-21 |
| UBA52 | 2.38E-24 | -0.29389 | 0.724 | 0.904 | 7.78E-20 |
| MGAT4A | 1.62E-27 | -0.29569 | 0.2 | 0.407 | 5.31E-23 |
| SPTBN1 | 1.05E-26 | -0.29702 | 0.092 | 0.236 | 3.45E-22 |
| RPLP1 | 4.36E-30 | -0.29753 | 0.945 | 0.984 | 1.43E-25 |
| GPX4 | 3.40E-29 | -0.29853 | 0.162 | 0.36 | 1.11E-24 |
| TMEM14B | 1.89E-26 | -0.29896 | 0.077 | 0.211 | 6.18E-22 |
| TSTD1 | 1.32E-25 | -0.3004 | 0.097 | 0.243 | 4.33E-21 |
| AES | 4.25E-31 | -0.30252 | 0.193 | 0.419 | 1.39E-26 |
| DRAP1 | 1.13E-27 | -0.30254 | 0.127 | 0.298 | 3.71E-23 |
| LRIG1 | 1.11E-22 | -0.30327 | 0.097 | 0.229 | 3.62E-18 |
| SEPW1 | 1.62E-27 | -0.3061 | 0.095 | 0.244 | 5.29E-23 |
| USP3 | 1.57E-28 | -0.30624 | 0.288 | 0.534 | 5.13E-24 |
| RPL10 | 1.63E-33 | -0.30774 | 0.934 | 0.976 | 5.34E-29 |
| RPS8 | 6.28E-29 | -0.30796 | 0.882 | 0.96 | 2.05E-24 |
| RPL37A | 1.92E-28 | -0.30823 | 0.75 | 0.916 | 6.28E-24 |
| RPL32 | 2.07E-32 | -0.30828 | 0.891 | 0.963 | 6.79E-28 |
| RPS9 | 4.82E-27 | -0.30897 | 0.788 | 0.931 | 1.58E-22 |
| YPEL3 | 1.11E-26 | -0.31007 | 0.096 | 0.242 | 3.62E-22 |
| MTMR6 | 1.86E-25 | -0.3104 | 0.06 | 0.178 | 6.08E-21 |
| S100A11 | 6.71E-29 | -0.31041 | 0.389 | 0.658 | 2.20E-24 |
| RPL9 | 7.63E-30 | -0.31046 | 0.82 | 0.946 | 2.50E-25 |
| SNX9 | 1.62E-24 | -0.3111 | 0.256 | 0.468 | 5.32E-20 |
| HNRNPA1 | 2.86E-26 | -0.31113 | 0.428 | 0.696 | 9.35E-22 |
| ARL6IP4 | 3.90E-31 | -0.31165 | 0.147 | 0.342 | 1.28E-26 |
| RPL7A | 4.75E-29 | -0.31216 | 0.813 | 0.937 | 1.55E-24 |
| NDUFS5 | 1.04E-27 | -0.31251 | 0.297 | 0.547 | 3.40E-23 |
| ITPKB | 3.65E-22 | -0.31266 | 0.144 | 0.298 | 1.19E-17 |
| MYL6 | 7.56E-25 | -0.31368 | 0.456 | 0.725 | 2.47E-20 |
| SLCO3A1 | 2.24E-27 | -0.31533 | 0.152 | 0.334 | 7.33E-23 |
| HINT1 | 6.40E-26 | -0.31553 | 0.368 | 0.636 | 2.09E-21 |
| EVI2A | 5.76E-28 | -0.31572 | 0.066 | 0.196 | 1.88E-23 |
| RPL18 | 1.63E-32 | -0.3161 | 0.839 | 0.95 | 5.33E-28 |
| METTL8 | 1.60E-24 | -0.31623 | 0.021 | 0.103 | 5.24E-20 |
| LAT | 1.38E-29 | -0.31817 | 0.067 | 0.203 | 4.53E-25 |
| CD5 | 4.51E-25 | -0.31979 | 0.051 | 0.161 | 1.48E-20 |
| CD48 | 1.15E-28 | -0.31983 | 0.31 | 0.565 | 3.76E-24 |
| MAP3K1 | 1.33E-23 | -0.32048 | 0.086 | 0.213 | 4.34E-19 |
| TLK1 | 4.05E-30 | -0.32068 | 0.212 | 0.432 | 1.33E-25 |
| ARPC3 | 6.87E-31 | -0.32151 | 0.277 | 0.54 | 2.25E-26 |
| PPP1R15A | 3.83E-25 | -0.32168 | 0.134 | 0.296 | 1.25E-20 |
| TMEM243 | 2.29E-30 | -0.32232 | 0.091 | 0.246 | 7.50E-26 |
| TSPO | 2.91E-30 | -0.32237 | 0.138 | 0.325 | 9.54E-26 |
| IL2RG | 7.84E-32 | -0.3228 | 0.179 | 0.396 | 2.57E-27 |
| EIF3K | 2.67E-28 | -0.32311 | 0.267 | 0.519 | 8.73E-24 |
| RPL22 | 4.76E-25 | -0.32331 | 0.62 | 0.851 | 1.56E-20 |
| RUNX1 | 1.88E-28 | -0.32357 | 0.287 | 0.528 | 6.16E-24 |
| TNIK | 5.52E-21 | -0.32452 | 0.351 | 0.581 | 1.81E-16 |
| FRMD4A | 1.26E-20 | -0.3255 | 0.049 | 0.143 | 4.14E-16 |
| TMEM173 | 2.01E-28 | -0.32577 | 0.058 | 0.184 | 6.60E-24 |
| EIF3E | 6.10E-32 | -0.32756 | 0.252 | 0.504 | 2.00E-27 |
| RPL13 | 1.36E-41 | -0.32919 | 0.95 | 0.987 | 4.44E-37 |
| CRTC3 | 1.01E-27 | -0.33054 | 0.077 | 0.215 | 3.32E-23 |
| ISCU | 1.95E-30 | -0.33185 | 0.1 | 0.263 | 6.37E-26 |
| AQP3 | 8.78E-29 | -0.33198 | 0.075 | 0.215 | 2.88E-24 |
| HIVEP1 | 1.41E-26 | -0.3374 | 0.134 | 0.301 | 4.62E-22 |
| PFKFB3 | 1.12E-28 | -0.33749 | 0.131 | 0.306 | 3.66E-24 |
| IRF2BP2 | 1.88E-27 | -0.33759 | 0.139 | 0.313 | 6.16E-23 |
| CIB1 | 1.13E-30 | -0.33802 | 0.207 | 0.431 | 3.68E-26 |
| RPS16 | 1.94E-35 | -0.33834 | 0.839 | 0.951 | 6.36E-31 |
| RPS2 | 2.93E-35 | -0.33838 | 0.889 | 0.964 | 9.59E-31 |
| KDSR | 2.81E-27 | -0.33898 | 0.064 | 0.191 | 9.21E-23 |
| FTH1 | 4.05E-29 | -0.33923 | 0.803 | 0.935 | 1.32E-24 |
| EEF1D | 4.91E-28 | -0.34063 | 0.549 | 0.807 | 1.61E-23 |
| NFATC1 | 1.02E-20 | -0.34109 | 0.044 | 0.135 | 3.35E-16 |
| TNFSF13B | 3.81E-25 | -0.3415 | 0.038 | 0.136 | 1.25E-20 |
| PRDX2 | 2.61E-34 | -0.34266 | 0.094 | 0.265 | 8.53E-30 |
| ZNF331 | 7.84E-24 | -0.34277 | 0.386 | 0.609 | 2.57E-19 |
| RPL27 | 7.17E-31 | -0.34287 | 0.666 | 0.889 | 2.35E-26 |
| RPLP2 | 1.61E-37 | -0.34298 | 0.873 | 0.965 | 5.27E-33 |
| ITM2A | 2.20E-30 | -0.34364 | 0.169 | 0.369 | 7.19E-26 |
| CYTIP | 1.16E-30 | -0.34382 | 0.276 | 0.533 | 3.79E-26 |
| HIST2H2AC | 7.95E-27 | -0.34404 | 0.07 | 0.199 | 2.60E-22 |
| CMSS1 | 9.82E-09 | -0.34441 | 0.229 | 0.339 | 0.000321 |
| ARPC1B | 4.14E-35 | -0.34485 | 0.171 | 0.396 | 1.35E-30 |
| TANK | 2.76E-30 | -0.34663 | 0.231 | 0.459 | 9.03E-26 |
| ESYT2 | 9.08E-33 | -0.34708 | 0.235 | 0.481 | 2.97E-28 |
| ANP32B | 2.29E-35 | -0.35062 | 0.182 | 0.413 | 7.49E-31 |
| GPSM3 | 6.24E-34 | -0.35177 | 0.096 | 0.268 | 2.04E-29 |
| LOH12CR1 | 3.83E-29 | -0.3525 | 0.159 | 0.35 | 1.25E-24 |
| SAMD12 | 7.77E-29 | -0.35613 | 0.091 | 0.24 | 2.54E-24 |
| NACA | 3.89E-31 | -0.35616 | 0.661 | 0.876 | 1.27E-26 |
| APRT | 3.05E-34 | -0.35649 | 0.177 | 0.403 | 9.99E-30 |
| FMN1 | 2.19E-23 | -0.35662 | 0.024 | 0.104 | 7.18E-19 |
| TAF4B | 1.03E-18 | -0.35721 | 0.056 | 0.148 | 3.36E-14 |
| MYL12B | 3.08E-26 | -0.35723 | 0.344 | 0.597 | 1.01E-21 |
| RGS10 | 4.33E-33 | -0.35723 | 0.123 | 0.31 | 1.42E-28 |
| SEPT6 | 1.07E-34 | -0.35728 | 0.275 | 0.54 | 3.49E-30 |
| YWHAB | 4.65E-31 | -0.35729 | 0.242 | 0.483 | 1.52E-26 |
| HIST1H3D | 1.01E-27 | -0.35776 | 0.068 | 0.198 | 3.30E-23 |
| EEF1B2 | 1.95E-28 | -0.35817 | 0.585 | 0.818 | 6.39E-24 |
| RPL15 | 6.59E-33 | -0.35854 | 0.809 | 0.918 | 2.16E-28 |
| SOCS3 | 4.26E-29 | -0.35882 | 0.088 | 0.236 | 1.39E-24 |
| CTLA4 | 2.79E-21 | -0.35926 | 0.039 | 0.127 | 9.13E-17 |
| ABCC1 | 4.18E-34 | -0.35993 | 0.157 | 0.37 | 1.37E-29 |
| RPSA | 1.24E-29 | -0.36171 | 0.656 | 0.875 | 4.07E-25 |
| RPL11 | 3.01E-39 | -0.36216 | 0.876 | 0.965 | 9.87E-35 |
| STAT3 | 3.32E-30 | -0.36227 | 0.284 | 0.532 | 1.09E-25 |
| S1PR1 | 3.91E-29 | -0.36273 | 0.044 | 0.159 | 1.28E-24 |
| EMP3 | 8.05E-33 | -0.36406 | 0.242 | 0.493 | 2.64E-28 |
| GMFG | 2.95E-35 | -0.36479 | 0.258 | 0.523 | 9.66E-31 |
| EID1 | 1.39E-35 | -0.36606 | 0.13 | 0.329 | 4.55E-31 |
| COTL1 | 4.36E-38 | -0.36614 | 0.127 | 0.331 | 1.43E-33 |
| RCAN3 | 3.10E-33 | -0.36777 | 0.204 | 0.434 | 1.01E-28 |
| ICAM2 | 2.69E-25 | -0.36841 | 0.035 | 0.131 | 8.81E-21 |
| SLC25A6 | 4.91E-31 | -0.36875 | 0.256 | 0.502 | 1.61E-26 |
| RPL19 | 6.85E-40 | -0.37031 | 0.88 | 0.961 | 2.24E-35 |
| ARL4C | 1.03E-28 | -0.37087 | 0.425 | 0.693 | 3.38E-24 |
| PHLDB3 | 6.55E-27 | -0.37193 | 0.03 | 0.125 | 2.14E-22 |
| DUSP16 | 2.88E-29 | -0.37241 | 0.256 | 0.49 | 9.41E-25 |
| RPL4 | 6.90E-30 | -0.37259 | 0.45 | 0.72 | 2.26E-25 |
| FAM89B | 2.91E-29 | -0.37351 | 0.059 | 0.187 | 9.51E-25 |
| RPS4X | 4.86E-39 | -0.37383 | 0.88 | 0.959 | 1.59E-34 |
| TMEM123 | 5.67E-33 | -0.37478 | 0.157 | 0.366 | 1.86E-28 |
| CYLD | 1.93E-30 | -0.3752 | 0.145 | 0.336 | 6.33E-26 |
| TAB2 | 3.30E-36 | -0.37546 | 0.173 | 0.396 | 1.08E-31 |
| ITGB1 | 3.36E-32 | -0.37591 | 0.211 | 0.436 | 1.10E-27 |
| LGALS8 | 6.38E-26 | -0.37846 | 0.055 | 0.17 | 2.09E-21 |
| GPCPD1 | 1.54E-24 | -0.37874 | 0.247 | 0.451 | 5.03E-20 |
| BATF | 2.98E-27 | -0.3789 | 0.076 | 0.21 | 9.75E-23 |
| TBL1X | 1.13E-28 | -0.37966 | 0.113 | 0.273 | 3.71E-24 |
| UXS1 | 4.86E-27 | -0.38263 | 0.059 | 0.179 | 1.59E-22 |
| SH3TC1 | 5.39E-32 | -0.38506 | 0.021 | 0.121 | 1.76E-27 |
| AHR | 8.41E-25 | -0.38665 | 0.123 | 0.274 | 2.75E-20 |
| TMEM66 | 7.75E-30 | -0.38793 | 0.579 | 0.832 | 2.54E-25 |
| TMA7 | 8.53E-32 | -0.38833 | 0.329 | 0.603 | 2.79E-27 |
| C11orf31 | 2.45E-30 | -0.39162 | 0.064 | 0.198 | 8.02E-26 |
| CD37 | 3.98E-38 | -0.39249 | 0.189 | 0.431 | 1.30E-33 |
| ARHGAP10 | 1.71E-27 | -0.39322 | 0.072 | 0.204 | 5.61E-23 |
| GLTSCR2 | 1.59E-37 | -0.39453 | 0.289 | 0.567 | 5.22E-33 |
| OCIAD2 | 4.79E-39 | -0.39515 | 0.164 | 0.398 | 1.57E-34 |
| MAST4 | 2.20E-29 | -0.39657 | 0.04 | 0.153 | 7.22E-25 |
| GNB2L1 | 2.56E-33 | -0.39736 | 0.656 | 0.862 | 8.38E-29 |
| RPL5 | 2.05E-35 | -0.40089 | 0.765 | 0.921 | 6.72E-31 |
| ANKRD12 | 2.05E-29 | -0.40299 | 0.534 | 0.792 | 6.71E-25 |
| LEF1 | 6.49E-26 | -0.4066 | 0.03 | 0.124 | 2.12E-21 |
| CNST | 2.94E-33 | -0.4118 | 0.089 | 0.251 | 9.62E-29 |
| RPS18 | 1.34E-48 | -0.41222 | 0.928 | 0.97 | 4.40E-44 |
| FURIN | 4.88E-31 | -0.41245 | 0.033 | 0.142 | 1.60E-26 |
| RPS20 | 1.24E-40 | -0.41418 | 0.716 | 0.909 | 4.05E-36 |
| KIAA1324L | 2.51E-30 | -0.41706 | 0.055 | 0.183 | 8.22E-26 |
| ACTB | 4.04E-39 | -0.41933 | 0.505 | 0.801 | 1.32E-34 |
| TNFRSF18 | 3.06E-30 | -0.41989 | 0.015 | 0.102 | 1.00E-25 |
| PELI1 | 1.08E-30 | -0.42162 | 0.081 | 0.229 | 3.54E-26 |
| RPS3A | 1.50E-44 | -0.42572 | 0.861 | 0.957 | 4.90E-40 |
| NDUFV2 | 2.22E-33 | -0.42617 | 0.151 | 0.353 | 7.27E-29 |
| RPL23 | 3.09E-38 | -0.42854 | 0.578 | 0.834 | 1.01E-33 |
| BTBD11 | 1.61E-23 | -0.43262 | 0.129 | 0.275 | 5.28E-19 |
| FYB | 4.30E-43 | -0.43381 | 0.301 | 0.599 | 1.41E-38 |
| HIST1H1C | 5.38E-35 | -0.43768 | 0.069 | 0.22 | 1.76E-30 |
| IRS2 | 6.65E-34 | -0.44001 | 0.083 | 0.241 | 2.18E-29 |
| TOMM7 | 5.91E-39 | -0.44026 | 0.397 | 0.695 | 1.94E-34 |
| IFITM2 | 5.09E-35 | -0.44115 | 0.257 | 0.507 | 1.67E-30 |
| MAF | 5.68E-28 | -0.44152 | 0.222 | 0.427 | 1.86E-23 |
| ZNRF1 | 1.09E-38 | -0.44325 | 0.079 | 0.25 | 3.58E-34 |
| MYL12A | 5.14E-38 | -0.44338 | 0.304 | 0.586 | 1.68E-33 |
| CD52 | 9.42E-39 | -0.44416 | 0.483 | 0.774 | 3.08E-34 |
| RORA | 1.59E-36 | -0.44461 | 0.471 | 0.754 | 5.21E-32 |
| NDNL2 | 2.04E-35 | -0.4452 | 0.09 | 0.258 | 6.67E-31 |
| RP11-219B17.1 | 1.39E-34 | -0.4453 | 0.194 | 0.417 | 4.56E-30 |
| IL32 | 3.51E-35 | -0.44628 | 0.396 | 0.681 | 1.15E-30 |
| RNASET2 | 4.72E-44 | -0.44691 | 0.154 | 0.396 | 1.55E-39 |
| PRKCA | 1.04E-32 | -0.4498 | 0.212 | 0.434 | 3.41E-28 |
| PTPN13 | 1.26E-33 | -0.45064 | 0.063 | 0.206 | 4.13E-29 |
| EEF1A1 | 2.08E-59 | -0.45196 | 0.934 | 0.986 | 6.80E-55 |
| GNAQ | 3.76E-39 | -0.45249 | 0.078 | 0.25 | 1.23E-34 |
| TRAF1 | 2.65E-37 | -0.45478 | 0.071 | 0.232 | 8.67E-33 |
| MFHAS1 | 4.38E-35 | -0.45544 | 0.047 | 0.181 | 1.43E-30 |
| S100A4 | 9.59E-34 | -0.45941 | 0.514 | 0.779 | 3.14E-29 |
| SERINC5 | 3.12E-41 | -0.45974 | 0.183 | 0.423 | 1.02E-36 |
| RPS6 | 6.21E-50 | -0.46107 | 0.846 | 0.956 | 2.03E-45 |
| EML4 | 5.48E-42 | -0.46182 | 0.55 | 0.832 | 1.79E-37 |
| SPOCK2 | 1.43E-46 | -0.46257 | 0.331 | 0.655 | 4.70E-42 |
| AP3M2 | 1.20E-39 | -0.46385 | 0.102 | 0.291 | 3.94E-35 |
| RPL29 | 1.03E-47 | -0.46621 | 0.74 | 0.924 | 3.37E-43 |
| MZT2A | 1.06E-43 | -0.46896 | 0.143 | 0.374 | 3.46E-39 |
| RPL7 | 3.58E-39 | -0.46916 | 0.616 | 0.857 | 1.17E-34 |
| JAK3 | 1.64E-38 | -0.46927 | 0.049 | 0.193 | 5.38E-34 |
| PLCL1 | 5.11E-30 | -0.46948 | 0.153 | 0.336 | 1.67E-25 |
| CD4 | 2.93E-36 | -0.47119 | 0.061 | 0.21 | 9.58E-32 |
| MAP3K4 | 1.06E-34 | -0.47505 | 0.111 | 0.289 | 3.47E-30 |
| GIMAP4 | 1.14E-39 | -0.47669 | 0.067 | 0.232 | 3.73E-35 |
| CD2 | 1.99E-41 | -0.4772 | 0.42 | 0.727 | 6.50E-37 |
| KLF2 | 8.01E-34 | -0.47954 | 0.332 | 0.577 | 2.62E-29 |
| MBP | 5.30E-42 | -0.48009 | 0.249 | 0.526 | 1.74E-37 |
| AGPAT5 | 1.56E-34 | -0.48092 | 0.048 | 0.179 | 5.12E-30 |
| RPL31 | 2.79E-44 | -0.48433 | 0.565 | 0.849 | 9.14E-40 |
| CLEC2D | 3.17E-50 | -0.48895 | 0.198 | 0.483 | 1.04E-45 |
| TNFAIP8 | 1.06E-45 | -0.49093 | 0.289 | 0.587 | 3.48E-41 |
| SLC2A3 | 8.24E-45 | -0.49111 | 0.276 | 0.562 | 2.70E-40 |
| TNFRSF25 | 5.46E-45 | -0.50139 | 0.077 | 0.264 | 1.79E-40 |
| TBC1D4 | 1.62E-40 | -0.50199 | 0.05 | 0.2 | 5.30E-36 |
| CORO1B | 1.40E-41 | -0.50278 | 0.053 | 0.209 | 4.57E-37 |
| SH3BGRL3 | 2.36E-47 | -0.50583 | 0.448 | 0.771 | 7.73E-43 |
| PFN1 | 6.49E-46 | -0.51131 | 0.398 | 0.714 | 2.13E-41 |
| LIMS1 | 1.68E-43 | -0.51213 | 0.143 | 0.37 | 5.50E-39 |
| DNPH1 | 2.20E-45 | -0.51233 | 0.029 | 0.169 | 7.21E-41 |
| CCR6 | 5.03E-43 | -0.51665 | 0.165 | 0.402 | 1.65E-38 |
| RPL3 | 2.66E-58 | -0.51719 | 0.856 | 0.958 | 8.72E-54 |
| JAZF1 | 2.85E-35 | -0.52694 | 0.201 | 0.422 | 9.34E-31 |
| RHOH | 5.51E-51 | -0.52826 | 0.286 | 0.602 | 1.81E-46 |
| RPS11 | 9.50E-59 | -0.53088 | 0.738 | 0.918 | 3.11E-54 |
| TIAM1 | 5.27E-33 | -0.53735 | 0.023 | 0.127 | 1.72E-28 |
| TPT1 | 1.92E-78 | -0.53761 | 0.927 | 0.981 | 6.30E-74 |
| LDHB | 5.19E-48 | -0.5412 | 0.168 | 0.423 | 1.70E-43 |
| HIST1H1E | 2.18E-44 | -0.5427 | 0.068 | 0.244 | 7.14E-40 |
| CD28 | 6.54E-50 | -0.54466 | 0.086 | 0.293 | 2.14E-45 |
| RPL13A | 4.31E-79 | -0.54696 | 0.883 | 0.978 | 1.41E-74 |
| ARID5B | 5.27E-59 | -0.54886 | 0.259 | 0.583 | 1.73E-54 |
| SELL | 5.70E-39 | -0.55242 | 0.029 | 0.153 | 1.87E-34 |
| BIRC3 | 1.14E-51 | -0.5629 | 0.241 | 0.538 | 3.72E-47 |
| FXYD5 | 2.15E-55 | -0.56807 | 0.226 | 0.533 | 7.03E-51 |
| PASK | 1.44E-48 | -0.56909 | 0.02 | 0.156 | 4.70E-44 |
| ACTG1 | 7.17E-53 | -0.57737 | 0.344 | 0.68 | 2.35E-48 |
| TPM4 | 1.07E-50 | -0.58942 | 0.146 | 0.396 | 3.50E-46 |
| RP4-678D15.1 | 5.71E-45 | -0.61303 | 0.006 | 0.111 | 1.87E-40 |
| CSGALNACT1 | 2.09E-42 | -0.62271 | 0.037 | 0.177 | 6.86E-38 |
| HIST1H1D | 9.36E-49 | -0.63499 | 0.1 | 0.307 | 3.06E-44 |
| GPRIN3 | 4.80E-59 | -0.63825 | 0.209 | 0.509 | 1.57E-54 |
| ADAM19 | 2.52E-43 | -0.63839 | 0.104 | 0.3 | 8.25E-39 |
| MAL | 1.79E-57 | -0.63857 | 0.016 | 0.165 | 5.86E-53 |
| CTSL | 7.46E-48 | -0.63969 | 0.02 | 0.153 | 2.44E-43 |
| RP11-712B9.2 | 5.59E-33 | -0.64496 | 0.041 | 0.162 | 1.83E-28 |
| ETV6 | 7.13E-58 | -0.66379 | 0.156 | 0.427 | 2.33E-53 |
| JUNB | 2.08E-54 | -0.67391 | 0.495 | 0.768 | 6.82E-50 |
| TMEM156 | 1.33E-55 | -0.67965 | 0.048 | 0.234 | 4.34E-51 |
| TNFRSF4 | 1.19E-58 | -0.68185 | 0.02 | 0.177 | 3.89E-54 |
| ZC3H12D | 5.78E-60 | -0.68786 | 0.052 | 0.251 | 1.89E-55 |
| CCR4 | 1.32E-59 | -0.69827 | 0.022 | 0.184 | 4.32E-55 |
| LDLRAD4 | 1.49E-62 | -0.71214 | 0.396 | 0.726 | 4.86E-58 |
| PGAP1 | 7.87E-34 | -0.71241 | 0.093 | 0.251 | 2.58E-29 |
| KLHL5 | 1.34E-50 | -0.72837 | 0.076 | 0.27 | 4.38E-46 |
| TMSB4X | 5.35E-128 | -0.78062 | 0.93 | 0.983 | 1.75E-123 |
| SESN3 | 3.77E-44 | -0.78904 | 0.075 | 0.25 | 1.23E-39 |
| PKIA | 1.27E-68 | -0.83319 | 0.026 | 0.213 | 4.16E-64 |
| PCBP3 | 3.31E-48 | -0.84734 | 0.022 | 0.158 | 1.08E-43 |
| CCR7 | 5.19E-86 | -0.86714 | 0.134 | 0.452 | 1.70E-81 |
| ANK3 | 2.01E-69 | -0.8686 | 0.193 | 0.5 | 6.59E-65 |
| GPR183 | 3.28E-87 | -0.91491 | 0.327 | 0.692 | 1.07E-82 |
| ICOS | 2.05E-102 | -1.05807 | 0.179 | 0.555 | 6.72E-98 |
| RPS26 | 6.26E-141 | -1.18672 | 0.443 | 0.818 | 2.05E-136 |
| LTB | 8.95E-153 | -1.52955 | 0.196 | 0.646 | 2.93E-148 |
| TSHZ2 | 7.43E-131 | -1.79594 | 0.043 | 0.366 | 2.43E-126 |
| FAAH2 | 1.09E-124 | -1.82941 | 0.074 | 0.412 | 3.57E-120 |

**Table S4: Top significant DE genes comparing expected tissue-resident and blood NK cells in PDAC tumor tissue, as identified by Originator.** Log2 fold change (avg_log2FC) is the averaged expression in expected tissue-resident compared to blood NK cells.

|  | **p_val** | **avg_log2FC** | **pct.1** | **pct.2** | **p_val_adj** |
| --- | --- | --- | --- | --- | --- |
| MLLT3 | 1.58E-08 | 0.919308 | 0.58 | 0.378 | 0.000517 |
| GZMK | 4.42E-17 | 0.856396 | 0.812 | 0.459 | 1.45E-12 |
| CCDC64 | 7.68E-09 | 0.825476 | 0.567 | 0.369 | 0.000251 |
| RGCC | 1.47E-10 | 0.792257 | 0.778 | 0.617 | 4.82E-06 |
| ZNF331 | 9.65E-08 | 0.632912 | 0.776 | 0.635 | 0.003159 |
| CD44 | 7.81E-08 | 0.528318 | 0.835 | 0.739 | 0.002555 |
| TMSB4X | 4.69E-08 | -0.35113 | 0.998 | 1 | 0.001535 |
| SH3BGRL3 | 1.85E-07 | -0.4571 | 0.724 | 0.865 | 0.006045 |
| PFN1 | 1.68E-07 | -0.47928 | 0.678 | 0.842 | 0.005509 |
| TMSB10 | 3.19E-08 | -0.48381 | 0.906 | 0.955 | 0.001044 |
| ARPC2 | 3.52E-10 | -0.60215 | 0.561 | 0.797 | 1.15E-05 |
| HCST | 1.66E-08 | -0.60324 | 0.516 | 0.725 | 0.000545 |
| MIR4435-1HG | 2.60E-08 | -0.60849 | 0.108 | 0.275 | 0.000853 |
| SYNGR1 | 6.01E-08 | -0.62617 | 0.02 | 0.113 | 0.001966 |
| CHST12 | 1.17E-08 | -0.645 | 0.082 | 0.239 | 0.000382 |
| MYL12A | 1.87E-10 | -0.71587 | 0.467 | 0.689 | 6.11E-06 |
| ACTG1 | 8.24E-09 | -0.73712 | 0.516 | 0.716 | 0.00027 |
| ITGB1 | 4.16E-08 | -0.73913 | 0.247 | 0.441 | 0.001361 |
| LYN | 6.91E-09 | -0.77626 | 0.027 | 0.14 | 0.000226 |
| CD63 | 1.91E-10 | -0.77783 | 0.194 | 0.419 | 6.24E-06 |
| KLRF1 | 4.79E-09 | -0.8157 | 0.027 | 0.14 | 0.000157 |
| CTB-91J4.1 | 7.64E-09 | -0.83219 | 0.02 | 0.122 | 0.00025 |
| EFHD2 | 1.38E-10 | -0.84341 | 0.2 | 0.432 | 4.52E-06 |
| ACTB | 4.75E-15 | -0.87106 | 0.69 | 0.856 | 1.55E-10 |
| AC092580.4 | 7.47E-11 | -0.87745 | 0.112 | 0.311 | 2.44E-06 |
| NCALD | 3.63E-10 | -0.92863 | 0.1 | 0.279 | 1.19E-05 |
| AGPAT4 | 1.02E-14 | -1.10832 | 0.112 | 0.351 | 3.33E-10 |
| HOPX | 1.16E-17 | -1.14243 | 0.063 | 0.297 | 3.81E-13 |
| KLRD1 | 1.25E-18 | -1.17805 | 0.106 | 0.378 | 4.08E-14 |
| LGALS1 | 3.51E-12 | -1.20687 | 0.216 | 0.455 | 1.15E-07 |
| KLRB1 | 1.46E-11 | -1.23468 | 0.076 | 0.257 | 4.77E-07 |
| LINGO2 | 4.02E-08 | -1.24521 | 0.016 | 0.104 | 0.001316 |
| TYROBP | 2.03E-16 | -1.24536 | 0.051 | 0.257 | 6.65E-12 |
| S100A4 | 1.10E-18 | -1.31244 | 0.365 | 0.676 | 3.61E-14 |
| FCGR3A | 2.51E-20 | -1.37518 | 0.02 | 0.221 | 8.20E-16 |
| NKG7 | 2.64E-40 | -1.46565 | 0.569 | 0.923 | 8.63E-36 |
| GZMH | 5.90E-35 | -1.55084 | 0.224 | 0.689 | 1.93E-30 |
| FGFBP2 | 7.79E-20 | -1.58029 | 0.041 | 0.27 | 2.55E-15 |
| PRF1 | 8.19E-24 | -1.62711 | 0.09 | 0.396 | 2.68E-19 |
| GZMB | 1.77E-41 | -2.31745 | 0.063 | 0.486 | 5.79E-37 |
| GNLY | 1.07E-41 | -2.91977 | 0.065 | 0.486 | 3.50E-37 |

**Table S5: Top significant DE genes comparing expected tissue-resident and blood macrophage cells in PDAC tumor tissue, as identified by Originator.** Log2 fold change (avg_log2FC) is the averaged expression in expected tissue-resident compared to blood macrophage cells.

|  | **p_val** | **avg_log2FC** | **pct.1** | **pct.2** | **p_val_adj** |
| --- | --- | --- | --- | --- | --- |
| EREG | 5.77E-12 | 1.587125 | 0.455 | 0.302 | 1.89E-07 |
| RND3 | 2.22E-19 | 1.572774 | 0.521 | 0.314 | 7.27E-15 |
| TNF | 1.48E-34 | 1.40944 | 0.658 | 0.314 | 4.84E-30 |
| CXCL3 | 1.52E-30 | 1.337026 | 0.819 | 0.644 | 4.99E-26 |
| LSAMP | 1.36E-20 | 1.330233 | 0.423 | 0.173 | 4.45E-16 |
| INHBA | 3.15E-13 | 1.308891 | 0.597 | 0.461 | 1.03E-08 |
| CCL20 | 1.11E-15 | 1.301512 | 0.496 | 0.292 | 3.62E-11 |
| FABP4 | 3.33E-21 | 1.269431 | 0.377 | 0.132 | 1.09E-16 |
| SGMS2 | 7.79E-22 | 1.191039 | 0.631 | 0.434 | 2.55E-17 |
| IL1B | 9.05E-19 | 1.185804 | 0.787 | 0.619 | 2.96E-14 |
| TEX14 | 3.65E-16 | 1.132171 | 0.736 | 0.551 | 1.19E-11 |
| ANXA1 | 1.47E-24 | 1.085911 | 0.941 | 0.901 | 4.80E-20 |
| ANKRD28 | 5.87E-32 | 1.056255 | 0.829 | 0.64 | 1.92E-27 |
| SVIL | 7.72E-19 | 1.00178 | 0.562 | 0.347 | 2.53E-14 |
| JUN | 3.14E-28 | 0.940978 | 0.966 | 0.892 | 1.03E-23 |
| DNAJB1 | 9.50E-27 | 0.934928 | 0.858 | 0.65 | 3.11E-22 |
| IER2 | 5.51E-25 | 0.932401 | 0.939 | 0.828 | 1.80E-20 |
| EGR1 | 1.30E-28 | 0.923217 | 0.895 | 0.67 | 4.27E-24 |
| B3GNT5 | 1.01E-26 | 0.923103 | 0.807 | 0.625 | 3.30E-22 |
| LRRC23 | 5.99E-11 | 0.915788 | 0.494 | 0.324 | 1.96E-06 |
| CITED2 | 4.70E-27 | 0.911896 | 0.778 | 0.585 | 1.54E-22 |
| KCNQ1OT1 | 2.90E-08 | 0.908648 | 0.345 | 0.209 | 0.00095 |
| PPP1R15A | 8.98E-35 | 0.905377 | 0.936 | 0.797 | 2.94E-30 |
| DENND5A | 9.17E-23 | 0.905237 | 0.848 | 0.788 | 3.00E-18 |
| IL1A | 3.74E-16 | 0.895255 | 0.565 | 0.366 | 1.22E-11 |
| BTG2 | 3.11E-29 | 0.893733 | 0.907 | 0.776 | 1.02E-24 |
| ADAM17 | 1.11E-35 | 0.885103 | 0.902 | 0.797 | 3.62E-31 |
| NFKBIZ | 1.34E-31 | 0.863318 | 0.941 | 0.756 | 4.40E-27 |
| OASL | 2.52E-24 | 0.861963 | 0.504 | 0.237 | 8.25E-20 |
| KLF4 | 5.26E-30 | 0.860441 | 0.936 | 0.797 | 1.72E-25 |
| CXCL2 | 1.37E-15 | 0.856468 | 0.807 | 0.655 | 4.48E-11 |
| DUSP2 | 3.87E-21 | 0.854534 | 0.802 | 0.599 | 1.27E-16 |
| FOS | 7.55E-24 | 0.838205 | 0.966 | 0.901 | 2.47E-19 |
| EIF4E | 3.43E-11 | 0.826687 | 0.67 | 0.561 | 1.12E-06 |
| HSPA1B | 5.91E-17 | 0.816766 | 0.746 | 0.554 | 1.93E-12 |
| CCL3 | 1.11E-09 | 0.810991 | 0.885 | 0.836 | 3.62E-05 |
| HSPA1A | 1.58E-13 | 0.804674 | 0.897 | 0.788 | 5.17E-09 |
| KB-1507C5.4 | 8.71E-10 | 0.794849 | 0.565 | 0.415 | 2.85E-05 |
| RAB7A | 3.56E-25 | 0.791889 | 0.973 | 0.921 | 1.17E-20 |
| UAP1 | 2.85E-20 | 0.791087 | 0.575 | 0.345 | 9.33E-16 |
| PMAIP1 | 3.82E-09 | 0.769728 | 0.562 | 0.424 | 0.000125 |
| SIK2 | 1.55E-07 | 0.768383 | 0.65 | 0.573 | 0.005088 |
| ATP2B1 | 3.08E-16 | 0.76712 | 0.922 | 0.896 | 1.01E-11 |
| PHLDA1 | 2.51E-23 | 0.756932 | 0.758 | 0.523 | 8.20E-19 |
| ADIPOR2 | 1.73E-10 | 0.755075 | 0.641 | 0.575 | 5.66E-06 |
| B4GALT1 | 9.45E-24 | 0.752053 | 0.902 | 0.818 | 3.10E-19 |
| DNAJA1 | 7.43E-25 | 0.751925 | 0.958 | 0.889 | 2.43E-20 |
| RGCC | 1.04E-07 | 0.743094 | 0.719 | 0.64 | 0.003401 |
| SEMA4A | 1.26E-09 | 0.741826 | 0.709 | 0.596 | 4.13E-05 |
| KLHL29 | 2.08E-18 | 0.741634 | 0.384 | 0.156 | 6.82E-14 |
| MMP19 | 4.95E-12 | 0.741109 | 0.689 | 0.541 | 1.62E-07 |
| MCF2L2 | 1.50E-29 | 0.731839 | 0.609 | 0.298 | 4.92E-25 |
| SHROOM3 | 2.05E-12 | 0.7092 | 0.174 | 0.046 | 6.71E-08 |
| RP11-701P16.5 | 3.90E-15 | 0.708258 | 0.484 | 0.281 | 1.28E-10 |
| ATP10A | 9.29E-16 | 0.707077 | 0.394 | 0.178 | 3.04E-11 |
| IL1RN | 1.59E-07 | 0.702058 | 0.575 | 0.465 | 0.00519 |
| FOSB | 4.48E-25 | 0.700165 | 0.985 | 0.853 | 1.47E-20 |
| C19orf59 | 1.71E-20 | 0.697194 | 0.535 | 0.274 | 5.59E-16 |
| RP11-779O18.3 | 1.58E-19 | 0.696941 | 0.733 | 0.479 | 5.18E-15 |
| ZSWIM6 | 8.47E-19 | 0.695827 | 0.985 | 0.941 | 2.77E-14 |
| DUSP1 | 9.54E-24 | 0.692711 | 0.973 | 0.899 | 3.12E-19 |
| DNAJA4 | 9.30E-17 | 0.684884 | 0.491 | 0.274 | 3.04E-12 |
| INTS6 | 5.41E-11 | 0.683984 | 0.672 | 0.529 | 1.77E-06 |
| IL8 | 2.08E-12 | 0.66944 | 0.902 | 0.772 | 6.81E-08 |
| CLIC4 | 1.57E-13 | 0.667582 | 0.885 | 0.779 | 5.14E-09 |
| HDAC9 | 1.17E-07 | 0.663882 | 0.34 | 0.209 | 0.003832 |
| KLF2 | 3.05E-14 | 0.656353 | 0.795 | 0.618 | 9.99E-10 |
| TNFAIP6 | 5.24E-11 | 0.656306 | 0.333 | 0.176 | 1.72E-06 |
| HBEGF | 1.91E-09 | 0.649177 | 0.697 | 0.607 | 6.25E-05 |
| MACC1 | 2.82E-07 | 0.63612 | 0.445 | 0.33 | 0.009235 |
| TCOF1 | 1.83E-11 | 0.624082 | 0.623 | 0.465 | 5.99E-07 |
| LINC00936 | 4.31E-19 | 0.618987 | 0.658 | 0.43 | 1.41E-14 |
| SERTAD1 | 5.26E-18 | 0.618646 | 0.724 | 0.529 | 1.72E-13 |
| CDKN1A | 8.61E-09 | 0.617549 | 0.68 | 0.575 | 0.000282 |
| FBXO11 | 1.43E-15 | 0.615544 | 0.878 | 0.785 | 4.69E-11 |
| RP11-58E21.3 | 6.46E-13 | 0.610365 | 0.32 | 0.147 | 2.12E-08 |
| NR4A1 | 5.00E-17 | 0.605293 | 0.785 | 0.609 | 1.64E-12 |
| UBB | 3.79E-21 | 0.601623 | 0.99 | 0.969 | 1.24E-16 |
| TREM1 | 6.89E-11 | 0.59822 | 0.697 | 0.615 | 2.26E-06 |
| ATF3 | 2.93E-20 | 0.598132 | 0.949 | 0.773 | 9.60E-16 |
| MIR155HG | 7.26E-09 | 0.592086 | 0.663 | 0.51 | 0.000238 |
| HSPH1 | 9.43E-08 | 0.590248 | 0.773 | 0.695 | 0.003088 |
| NFKBIA | 3.63E-17 | 0.589396 | 0.993 | 0.919 | 1.19E-12 |
| PPP1R10 | 2.82E-15 | 0.582846 | 0.724 | 0.544 | 9.23E-11 |
| TNFRSF10B | 2.21E-09 | 0.576509 | 0.579 | 0.439 | 7.22E-05 |
| H3F3B | 1.64E-25 | 0.573426 | 0.995 | 0.979 | 5.38E-21 |
| TUBB4B | 1.06E-11 | 0.572193 | 0.814 | 0.708 | 3.46E-07 |
| ABL2 | 4.67E-13 | 0.571107 | 0.883 | 0.806 | 1.53E-08 |
| EGR2 | 5.35E-10 | 0.569174 | 0.667 | 0.498 | 1.75E-05 |
| TSC22D2 | 4.97E-17 | 0.56705 | 0.897 | 0.742 | 1.63E-12 |
| OTUD1 | 1.22E-13 | 0.566881 | 0.653 | 0.477 | 3.99E-09 |
| KCNE1 | 1.24E-11 | 0.562304 | 0.553 | 0.361 | 4.08E-07 |
| TCF7L2 | 1.02E-14 | 0.56188 | 0.775 | 0.639 | 3.35E-10 |
| STK17B | 5.56E-16 | 0.550407 | 0.638 | 0.437 | 1.82E-11 |
| RP11-24F11.2 | 9.95E-12 | 0.548366 | 0.391 | 0.227 | 3.26E-07 |
| IGFBP2 | 5.48E-08 | 0.543188 | 0.433 | 0.287 | 0.001793 |
| CCDC18 | 2.78E-09 | 0.541281 | 0.455 | 0.299 | 9.10E-05 |
| UBE2R2 | 2.45E-09 | 0.53771 | 0.773 | 0.708 | 8.01E-05 |
| NFE2L3 | 1.81E-09 | 0.536286 | 0.499 | 0.332 | 5.94E-05 |
| RP11-598F7.3 | 8.00E-09 | 0.534108 | 0.423 | 0.29 | 0.000262 |

**Table S6: Top significant DE genes comparing expected tissue-resident and blood monocytes in PDAC tumor tissue, as identified by Originator.** Log2 fold change (avg_log2FC) is the averaged expression in expected tissue-resident compared to blood monocytes.

|  | p_val | avg_log2FC | pct.1 | pct.2 | p_val_adj |
| --- | --- | --- | --- | --- | --- |
| CPA3 | 3.10E-15 | 1.760257 | 0.2 | 0 | 1.02E-10 |
| KRT5 | 1.46E-19 | 1.741882 | 0.4 | 0.003 | 4.78E-15 |
| CLEC4C | 2.42E-12 | 1.737032 | 0.4 | 0.01 | 7.93E-08 |
| PACSIN1 | 1.82E-10 | 1.715609 | 0.4 | 0.013 | 5.95E-06 |
| LILRA5 | 6.18E-09 | 1.563429 | 0.8 | 0.08 | 0.000202 |
| SCT | 1.82E-10 | 1.526098 | 0.4 | 0.013 | 5.95E-06 |
| TPSAB1 | 3.51E-08 | 1.365937 | 0.2 | 0.003 | 0.001149 |
| VASH2 | 3.35E-12 | 1.357507 | 0.4 | 0.01 | 1.10E-07 |
| MAP1A | 5.07E-08 | 1.336734 | 0.4 | 0.019 | 0.001658 |
| CERS4 | 1.49E-09 | 1.221591 | 0.6 | 0.038 | 4.86E-05 |
| LILRA1 | 4.02E-09 | 1.124013 | 0.4 | 0.016 | 0.000131 |
| ASIP | 4.13E-08 | 1.101175 | 0.4 | 0.019 | 0.001352 |
| CTD-2023N9.1 | 4.62E-29 | 1.029141 | 0.4 | 0 | 1.51E-24 |
| BSPRY | 8.53E-20 | 0.847571 | 0.4 | 0.003 | 2.79E-15 |
| TAL1 | 3.10E-15 | 0.844676 | 0.2 | 0 | 1.02E-10 |
| AC097495.2 | 3.10E-15 | 0.844676 | 0.2 | 0 | 1.02E-10 |
| LCN6.1 | 3.10E-15 | 0.844676 | 0.2 | 0 | 1.02E-10 |
| RP11-573G6.10 | 3.10E-15 | 0.844676 | 0.2 | 0 | 1.02E-10 |
| OR10A4 | 3.10E-15 | 0.844676 | 0.2 | 0 | 1.02E-10 |
| EMID1 | 3.10E-15 | 0.844676 | 0.2 | 0 | 1.02E-10 |
| PPARGC1A | 3.51E-08 | 0.841151 | 0.2 | 0.003 | 0.001149 |
| F2RL3 | 3.51E-08 | 0.836686 | 0.2 | 0.003 | 0.001149 |
| AC074117.10 | 4.02E-09 | 0.831836 | 0.4 | 0.016 | 0.000131 |
| SMPD3 | 3.94E-12 | 0.819265 | 0.4 | 0.01 | 1.29E-07 |
| LRRC36 | 3.51E-08 | 0.812328 | 0.2 | 0.003 | 0.001149 |
| PTGES | 5.73E-15 | 0.568231 | 0.4 | 0.006 | 1.87E-10 |
| N4BP3 | 5.07E-08 | 0.555367 | 0.4 | 0.019 | 0.001658 |
| AC062017.1 | 9.27E-08 | 0.537653 | 0.4 | 0.019 | 0.003033 |
| RIMKLA | 3.10E-15 | 0.509674 | 0.2 | 0 | 1.02E-10 |
| RP11-161D15.3 | 3.10E-15 | 0.509674 | 0.2 | 0 | 1.02E-10 |
| RP11-844P9.2 | 3.10E-15 | 0.509674 | 0.2 | 0 | 1.02E-10 |
| GATSL1 | 3.10E-15 | 0.509674 | 0.2 | 0 | 1.02E-10 |
| ATE1-AS1 | 3.10E-15 | 0.509674 | 0.2 | 0 | 1.02E-10 |
| ADAMTSL3 | 3.10E-15 | 0.509674 | 0.2 | 0 | 1.02E-10 |
| C19orf84 | 3.10E-15 | 0.509674 | 0.2 | 0 | 1.02E-10 |
| AC004837.5 | 3.51E-08 | 0.507871 | 0.2 | 0.003 | 0.001149 |
| RP11-783K16.14 | 3.51E-08 | 0.507136 | 0.2 | 0.003 | 0.001149 |
| RP11-530C5.1 | 3.51E-08 | 0.507107 | 0.2 | 0.003 | 0.001149 |
| RP11-473O4.5 | 3.51E-08 | 0.506426 | 0.2 | 0.003 | 0.001149 |
| RP11-275F13.1 | 3.51E-08 | 0.505324 | 0.2 | 0.003 | 0.001149 |
| RP11-134O21.1 | 3.51E-08 | 0.504989 | 0.2 | 0.003 | 0.001149 |
| FAM227A | 3.51E-08 | 0.501919 | 0.2 | 0.003 | 0.001149 |
| FAM69B | 3.51E-08 | 0.500147 | 0.2 | 0.003 | 0.001149 |
| RP11-71G12.1 | 4.30E-08 | 0.494773 | 0.2 | 0.003 | 0.001407 |
| AC011893.3 | 3.10E-15 | 0.464982 | 0.2 | 0 | 1.02E-10 |
| RP11-33O4.1 | 3.10E-15 | 0.464982 | 0.2 | 0 | 1.02E-10 |
| GP5 | 3.10E-15 | 0.464982 | 0.2 | 0 | 1.02E-10 |
| SBSPON | 3.10E-15 | 0.464982 | 0.2 | 0 | 1.02E-10 |
| RP11-388P9.2 | 3.10E-15 | 0.464982 | 0.2 | 0 | 1.02E-10 |
| KCNA5 | 3.10E-15 | 0.464982 | 0.2 | 0 | 1.02E-10 |
| CTD-2292M16.8 | 3.10E-15 | 0.464982 | 0.2 | 0 | 1.02E-10 |
| LTK | 3.10E-15 | 0.464982 | 0.2 | 0 | 1.02E-10 |
| MAG | 3.10E-15 | 0.464982 | 0.2 | 0 | 1.02E-10 |
| AP001439.2 | 3.10E-15 | 0.464982 | 0.2 | 0 | 1.02E-10 |
| RP11-388M20.1 | 3.51E-08 | 0.461734 | 0.2 | 0.003 | 0.001149 |
| ARHGEF16 | 3.51E-08 | 0.461418 | 0.2 | 0.003 | 0.001149 |
| RP5-1031D4.2 | 3.51E-08 | 0.461243 | 0.2 | 0.003 | 0.001149 |
| WNT10A | 3.51E-08 | 0.457512 | 0.2 | 0.003 | 0.001149 |
| PPFIA4 | 3.10E-15 | 0.318361 | 0.2 | 0 | 1.02E-10 |
| RP11-31K23.2 | 3.51E-08 | 0.316219 | 0.2 | 0.003 | 0.001149 |
| RP11-505P4.6 | 3.10E-15 | 0.316085 | 0.2 | 0 | 1.02E-10 |
| AC020629.1 | 3.10E-15 | 0.316085 | 0.2 | 0 | 1.02E-10 |
| PRKD1 | 3.51E-08 | 0.315562 | 0.2 | 0.003 | 0.001149 |
| KCNH3 | 3.51E-08 | 0.314667 | 0.2 | 0.003 | 0.001149 |
| CTD-2267D19.3 | 3.51E-08 | 0.314257 | 0.2 | 0.003 | 0.001149 |
| AC007970.1 | 3.51E-08 | 0.313992 | 0.2 | 0.003 | 0.001149 |
| RP11-290L1.3 | 3.51E-08 | 0.313951 | 0.2 | 0.003 | 0.001149 |
| CLMP | 3.51E-08 | 0.313762 | 0.2 | 0.003 | 0.001149 |
| SHPK | 3.51E-08 | 0.312722 | 0.2 | 0.003 | 0.001149 |
| SEZ6 | 3.51E-08 | 0.311999 | 0.2 | 0.003 | 0.001149 |
| SERPINB10 | 4.30E-08 | 0.306728 | 0.2 | 0.003 | 0.001407 |

**Table S7: Top significant DE genes comparing fetal and maternal fibroblast type 1 cells in placenta tissue, as identified by Originator.** Log2 fold change (avg_log2FC) is the averaged expression in the fetal compared to maternal fibroblast type 1.

|  | **p_val** | **avg_log2FC** | **pct.1** | **pct.2** | **p_val_adj** |
| --- | --- | --- | --- | --- | --- |
| EGFL6 | 1.59E-243 | 4.429656 | 0.961 | 0.125 | 3.51E-239 |
| TCF21 | 1.86E-197 | 4.315008 | 0.868 | 0.089 | 4.13E-193 |
| GPC3 | 3.45E-235 | 4.239064 | 0.946 | 0.114 | 7.65E-231 |
| PLA2G2A | 8.73E-156 | 4.072042 | 0.79 | 0.105 | 1.93E-151 |
| CD36 | 5.40E-180 | 3.637041 | 0.831 | 0.093 | 1.20E-175 |
| WNT2 | 1.56E-154 | 3.371118 | 0.753 | 0.039 | 3.46E-150 |
| HSD17B2 | 1.30E-197 | 3.30591 | 0.883 | 0.155 | 2.88E-193 |
| DLK1 | 1.10E-162 | 3.288633 | 0.817 | 0.098 | 2.43E-158 |
| C7 | 1.69E-138 | 3.251991 | 0.748 | 0.095 | 3.75E-134 |
| HAPLN1 | 3.37E-91 | 3.108324 | 0.545 | 0.011 | 7.47E-87 |
| RARRES2 | 2.42E-143 | 3.066998 | 0.757 | 0.091 | 5.37E-139 |
| PITX2 | 7.50E-145 | 3.029137 | 0.724 | 0.03 | 1.66E-140 |
| PHACTR2 | 3.62E-166 | 2.886097 | 0.827 | 0.202 | 8.02E-162 |
| HGF | 3.26E-101 | 2.754256 | 0.606 | 0.05 | 7.24E-97 |
| SEPP1 | 1.24E-175 | 2.721012 | 0.936 | 0.673 | 2.75E-171 |
| MEG3 | 1.40E-212 | 2.720558 | 0.981 | 0.659 | 3.10E-208 |
| SERPINF1 | 1.88E-171 | 2.709533 | 0.929 | 0.641 | 4.18E-167 |
| PMP22 | 2.47E-162 | 2.605238 | 0.864 | 0.352 | 5.47E-158 |
| COL3A1 | 4.15E-149 | 2.567016 | 0.891 | 0.475 | 9.19E-145 |
| CYTL1 | 7.92E-54 | 2.556446 | 0.378 | 0.005 | 1.75E-49 |
| C12orf39 | 3.08E-56 | 2.458759 | 0.396 | 0.009 | 6.83E-52 |
| CNN3 | 7.64E-148 | 2.448055 | 0.825 | 0.227 | 1.69E-143 |
| RPS23 | 1.09E-226 | 2.393955 | 0.997 | 0.827 | 2.42E-222 |
| S100A10 | 1.78E-160 | 2.389769 | 0.891 | 0.373 | 3.95E-156 |
| FGF7 | 7.51E-54 | 2.342571 | 0.488 | 0.152 | 1.67E-49 |
| TAGLN2 | 1.63E-120 | 2.290443 | 0.8 | 0.395 | 3.61E-116 |
| GPX3 | 2.58E-108 | 2.251919 | 0.812 | 0.361 | 5.72E-104 |
| ENPP2 | 2.21E-112 | 2.238685 | 0.77 | 0.33 | 4.90E-108 |
| TFPI2 | 1.04E-32 | 2.16592 | 0.321 | 0.061 | 2.30E-28 |
| PDPN | 1.89E-99 | 2.126445 | 0.791 | 0.573 | 4.20E-95 |
| CADM3 | 2.79E-65 | 2.109031 | 0.438 | 0.011 | 6.18E-61 |
| MATN2 | 1.01E-84 | 2.108842 | 0.632 | 0.216 | 2.25E-80 |
| AKR1B1 | 8.04E-72 | 2.067901 | 0.742 | 0.58 | 1.78E-67 |
| ANGPTL1 | 3.19E-85 | 2.058844 | 0.633 | 0.18 | 7.08E-81 |
| TMEM176A | 3.96E-80 | 2.052475 | 0.646 | 0.223 | 8.78E-76 |
| RPL23A | 4.90E-210 | 2.044697 | 0.997 | 0.814 | 1.09E-205 |
| PLAC1 | 9.13E-62 | 2.017424 | 0.424 | 0.014 | 2.02E-57 |
| SORBS2 | 1.47E-75 | 1.993688 | 0.513 | 0.043 | 3.26E-71 |
| RPL27A | 1.57E-213 | 1.992075 | 0.998 | 0.88 | 3.49E-209 |
| RPS27 | 6.63E-175 | 1.947134 | 0.986 | 0.752 | 1.47E-170 |
| RPL36A | 2.07E-123 | 1.85864 | 0.845 | 0.45 | 4.59E-119 |
| RPL21 | 1.80E-184 | 1.858082 | 0.994 | 0.814 | 4.00E-180 |
| RPL26 | 1.14E-191 | 1.844048 | 0.997 | 0.857 | 2.53E-187 |
| RPL34 | 1.75E-180 | 1.843375 | 0.995 | 0.83 | 3.87E-176 |
| RPS25 | 9.10E-160 | 1.827714 | 0.964 | 0.645 | 2.02E-155 |
| RPS28 | 3.29E-152 | 1.816583 | 0.949 | 0.695 | 7.30E-148 |
| BMP5 | 9.49E-59 | 1.802208 | 0.414 | 0.016 | 2.10E-54 |
| RPS15A | 1.64E-178 | 1.793451 | 0.99 | 0.782 | 3.62E-174 |
| RPL7 | 7.21E-193 | 1.791191 | 0.997 | 0.868 | 1.60E-188 |
| BMP4 | 6.32E-52 | 1.77684 | 0.424 | 0.068 | 1.40E-47 |
| TMEM176B | 1.23E-87 | 1.759691 | 0.776 | 0.386 | 2.72E-83 |
| RPS3A | 8.65E-179 | 1.747539 | 0.991 | 0.825 | 1.92E-174 |
| RPL31 | 7.48E-169 | 1.733673 | 0.993 | 0.789 | 1.66E-164 |
| RPL38 | 9.06E-119 | 1.720346 | 0.863 | 0.516 | 2.01E-114 |
| CD9 | 4.82E-76 | 1.714701 | 0.653 | 0.218 | 1.07E-71 |
| NUPR1 | 6.29E-97 | 1.695084 | 0.85 | 0.673 | 1.39E-92 |
| RPS14 | 9.13E-174 | 1.663032 | 0.998 | 0.907 | 2.02E-169 |
| FRZB | 2.03E-30 | 1.655166 | 0.31 | 0.057 | 4.51E-26 |
| COL15A1 | 1.55E-43 | 1.653874 | 0.46 | 0.164 | 3.44E-39 |
| RPS4Y1 | 4.71E-14 | 1.652484 | 0.314 | 0.193 | 1.05E-09 |
| RPS27A | 1.26E-175 | 1.651167 | 0.996 | 0.902 | 2.79E-171 |
| RPS29 | 2.65E-88 | 1.649661 | 0.754 | 0.373 | 5.88E-84 |
| RPL30 | 1.02E-159 | 1.646104 | 0.99 | 0.791 | 2.26E-155 |
| HNRNPA1 | 1.96E-107 | 1.633338 | 0.838 | 0.625 | 4.34E-103 |
| RPS24 | 5.74E-160 | 1.631131 | 0.986 | 0.805 | 1.27E-155 |
| FIBIN | 6.17E-42 | 1.624765 | 0.333 | 0.02 | 1.37E-37 |
| UQCRB | 1.13E-54 | 1.618989 | 0.642 | 0.38 | 2.49E-50 |
| GNG11 | 1.96E-32 | 1.587127 | 0.532 | 0.339 | 4.34E-28 |
| RPL24 | 2.99E-160 | 1.577585 | 0.981 | 0.82 | 6.63E-156 |
| RPL14 | 1.25E-164 | 1.569504 | 0.991 | 0.877 | 2.77E-160 |
| SEPT7 | 6.35E-75 | 1.569425 | 0.731 | 0.505 | 1.41E-70 |
| SPARC | 1.70E-171 | 1.564863 | 0.99 | 0.92 | 3.77E-167 |
| RPLP2 | 7.57E-162 | 1.545529 | 0.997 | 0.886 | 1.68E-157 |
| EIF3E | 8.54E-76 | 1.543778 | 0.759 | 0.548 | 1.89E-71 |
| NPM1 | 1.01E-120 | 1.539533 | 0.892 | 0.716 | 2.23E-116 |
| BST2 | 6.73E-49 | 1.514151 | 0.584 | 0.27 | 1.49E-44 |
| ELN | 2.76E-38 | 1.502515 | 0.342 | 0.048 | 6.12E-34 |
| RPL15 | 7.38E-176 | 1.49784 | 0.998 | 0.964 | 1.64E-171 |
| RPS8 | 5.67E-154 | 1.496914 | 0.997 | 0.893 | 1.26E-149 |
| PCOLCE2 | 4.58E-45 | 1.496335 | 0.371 | 0.036 | 1.01E-40 |
| PLP2 | 1.86E-55 | 1.493829 | 0.652 | 0.436 | 4.13E-51 |
| PTGDS | 2.85E-52 | 1.490433 | 0.863 | 0.859 | 6.33E-48 |
| CFL2 | 3.10E-41 | 1.4873 | 0.543 | 0.32 | 6.87E-37 |
| ID3 | 3.36E-21 | 1.467823 | 0.474 | 0.323 | 7.45E-17 |
| EIF4A2 | 1.41E-92 | 1.453702 | 0.866 | 0.759 | 3.13E-88 |
| HMGB2 | 2.07E-39 | 1.440726 | 0.484 | 0.223 | 4.58E-35 |
| RPS3 | 7.07E-158 | 1.437167 | 0.995 | 0.936 | 1.57E-153 |
| RPS20 | 2.22E-134 | 1.436909 | 0.972 | 0.784 | 4.92E-130 |
| TPT1 | 1.25E-147 | 1.432744 | 0.998 | 0.957 | 2.78E-143 |
| OLFML3 | 4.91E-36 | 1.412725 | 0.662 | 0.559 | 1.09E-31 |
| RPS13 | 1.88E-142 | 1.410261 | 0.989 | 0.85 | 4.16E-138 |
| RPL9 | 4.10E-150 | 1.404303 | 0.989 | 0.893 | 9.10E-146 |
| TSC22D1 | 2.78E-31 | 1.403521 | 0.573 | 0.42 | 6.17E-27 |
| RPL37A | 3.41E-112 | 1.401321 | 0.935 | 0.73 | 7.57E-108 |
| RPS7 | 2.08E-151 | 1.399044 | 0.99 | 0.907 | 4.60E-147 |
| RPS15 | 1.23E-150 | 1.395557 | 0.996 | 0.916 | 2.73E-146 |
| RPL32 | 2.78E-142 | 1.394387 | 0.998 | 0.925 | 6.17E-138 |
| LSP1 | 1.92E-33 | 1.390285 | 0.342 | 0.08 | 4.25E-29 |
| SERPINE2 | 1.11E-44 | 1.38508 | 0.581 | 0.291 | 2.46E-40 |
| RPL35A | 1.75E-140 | 1.374752 | 0.99 | 0.855 | 3.87E-136 |

**Table S8: Top significant DE genes comparing fetal and maternal fibroblast type 2 cells in placenta tissue, as identified by Originator.** Log2 fold change (avg_log2FC) is the averaged expression in the fetal compared to maternal fibroblast type 2.

|  | **p_val** | **avg_log2FC** | **pct.1** | **pct.2** | **p_val_adj** |
| --- | --- | --- | --- | --- | --- |
| EGFL6 | 1.15E-97 | 2.618358 | 0.28 | 0.05 | 2.56E-93 |
| TCF21 | 2.16E-114 | 2.612331 | 0.271 | 0.032 | 4.79E-110 |
| GPC3 | 9.91E-104 | 2.512707 | 0.287 | 0.048 | 2.20E-99 |
| TFPI2 | 1.58E-46 | 2.028858 | 0.145 | 0.026 | 3.50E-42 |
| DLK1 | 2.05E-91 | 1.895907 | 0.273 | 0.051 | 4.55E-87 |
| COL3A1 | 1.31E-39 | 1.799687 | 0.442 | 0.273 | 2.91E-35 |
| MEG3 | 8.54E-56 | 1.785014 | 0.721 | 0.573 | 1.89E-51 |
| CD36 | 1.01E-93 | 1.757117 | 0.257 | 0.04 | 2.23E-89 |
| S100A10 | 6.75E-180 | 1.60851 | 0.782 | 0.354 | 1.50E-175 |
| HSD17B2 | 6.80E-73 | 1.600796 | 0.24 | 0.05 | 1.51E-68 |
| IGF2 | 4.09E-77 | 1.595697 | 0.885 | 0.745 | 9.07E-73 |
| IGFBP3 | 1.74E-132 | 1.491305 | 0.946 | 0.806 | 3.85E-128 |
| FN1 | 1.06E-108 | 1.485688 | 0.862 | 0.691 | 2.35E-104 |
| HAPLN1 | 2.43E-68 | 1.3866 | 0.128 | 0.005 | 5.39E-64 |
| PITX2 | 3.98E-119 | 1.384857 | 0.211 | 0.007 | 8.82E-115 |
| EPYC | 9.74E-99 | 1.382534 | 0.401 | 0.12 | 2.16E-94 |
| SERPINE2 | 1.19E-78 | 1.346266 | 0.445 | 0.18 | 2.64E-74 |
| SPARC | 2.66E-125 | 1.28373 | 0.964 | 0.811 | 5.89E-121 |
| PLA2G2A | 8.61E-23 | 1.26497 | 0.213 | 0.103 | 1.91E-18 |
| TAC3 | 1.77E-58 | 1.263857 | 0.444 | 0.213 | 3.92E-54 |
| WNT2 | 5.83E-64 | 1.259141 | 0.208 | 0.041 | 1.29E-59 |
| HGF | 3.71E-80 | 1.238979 | 0.174 | 0.015 | 8.23E-76 |
| PDPN | 4.69E-15 | 1.230947 | 0.549 | 0.486 | 1.04E-10 |
| C7 | 5.50E-51 | 1.230843 | 0.193 | 0.046 | 1.22E-46 |
| CYTL1 | 1.03E-53 | 1.219926 | 0.1 | 0.004 | 2.29E-49 |
| ENPP2 | 4.13E-50 | 1.171042 | 0.308 | 0.124 | 9.15E-46 |
| COL1A1 | 8.28E-32 | 1.14892 | 0.661 | 0.541 | 1.84E-27 |
| PHACTR2 | 1.05E-24 | 1.127201 | 0.369 | 0.248 | 2.32E-20 |
| CSH1 | 2.13E-29 | 1.125288 | 0.645 | 0.532 | 4.72E-25 |
| PRG2 | 4.21E-75 | 1.089949 | 0.572 | 0.325 | 9.33E-71 |
| RARRES2 | 1.68E-11 | 1.076482 | 0.23 | 0.153 | 3.72E-07 |
| IGFBP1 | 5.03E-13 | 1.076354 | 0.755 | 0.847 | 1.12E-08 |
| MATN2 | 4.80E-35 | 0.976729 | 0.23 | 0.093 | 1.06E-30 |
| TIMP2 | 2.72E-65 | 0.972391 | 0.819 | 0.783 | 6.02E-61 |
| PPDPF | 2.70E-126 | 0.966404 | 0.94 | 0.946 | 5.99E-122 |
| PMP22 | 2.37E-09 | 0.964084 | 0.422 | 0.382 | 5.26E-05 |
| COL1A2 | 2.51E-14 | 0.956048 | 0.417 | 0.333 | 5.56E-10 |
| GNAS | 4.56E-36 | 0.956025 | 0.727 | 0.673 | 1.01E-31 |
| TIMP3 | 1.26E-38 | 0.951015 | 0.929 | 0.969 | 2.78E-34 |
| TMEM176B | 5.65E-34 | 0.937679 | 0.564 | 0.365 | 1.25E-29 |
| KRT18 | 1.96E-23 | 0.934884 | 0.575 | 0.485 | 4.35E-19 |
| CD9 | 2.35E-63 | 0.926787 | 0.32 | 0.109 | 5.21E-59 |
| CNN3 | 1.70E-15 | 0.924418 | 0.345 | 0.248 | 3.76E-11 |
| TMEM98 | 7.11E-88 | 0.884145 | 0.821 | 0.705 | 1.58E-83 |
| TAGLN2 | 1.10E-32 | 0.868028 | 0.669 | 0.574 | 2.44E-28 |
| JAM2 | 7.44E-54 | 0.836138 | 0.573 | 0.396 | 1.65E-49 |
| ADAMTS1 | 8.64E-35 | 0.800778 | 0.761 | 0.686 | 1.91E-30 |
| KCNQ1OT1 | 3.23E-54 | 0.777925 | 0.587 | 0.378 | 7.15E-50 |
| CRLF1 | 5.44E-86 | 0.769451 | 0.361 | 0.106 | 1.21E-81 |
| CITED2 | 1.72E-22 | 0.737314 | 0.647 | 0.59 | 3.82E-18 |
| KRT8 | 2.11E-12 | 0.731913 | 0.391 | 0.312 | 4.67E-08 |
| CLU | 3.36E-38 | 0.730333 | 0.722 | 0.608 | 7.45E-34 |
| IL1RL1 | 2.00E-11 | 0.729334 | 0.453 | 0.369 | 4.43E-07 |
| C12orf39 | 2.18E-55 | 0.723933 | 0.106 | 0.005 | 4.84E-51 |
| RPS4Y1 | 2.55E-31 | 0.723712 | 0.212 | 0.083 | 5.66E-27 |
| CRISPLD2 | 4.10E-52 | 0.722449 | 0.778 | 0.676 | 9.08E-48 |
| VTN | 2.37E-14 | 0.720926 | 0.34 | 0.25 | 5.24E-10 |
| TGFBR1 | 3.40E-72 | 0.715112 | 0.385 | 0.146 | 7.54E-68 |
| ATP2B4 | 9.49E-33 | 0.708239 | 0.622 | 0.533 | 2.10E-28 |
| PAPPA | 1.11E-24 | 0.703654 | 0.619 | 0.533 | 2.46E-20 |
| PPIC | 4.13E-46 | 0.696404 | 0.686 | 0.52 | 9.17E-42 |
| TMEM176A | 3.69E-14 | 0.685517 | 0.435 | 0.318 | 8.18E-10 |
| TGFBI | 2.88E-16 | 0.678673 | 0.403 | 0.293 | 6.37E-12 |
| VEGFA | 1.51E-11 | 0.677592 | 0.409 | 0.326 | 3.35E-07 |
| PRUNE2 | 5.06E-45 | 0.677505 | 0.572 | 0.376 | 1.12E-40 |
| AOC1 | 1.09E-15 | 0.677382 | 0.357 | 0.252 | 2.41E-11 |
| PAPPA2 | 2.09E-17 | 0.673445 | 0.188 | 0.095 | 4.63E-13 |
| HTRA1 | 1.08E-28 | 0.672958 | 0.525 | 0.391 | 2.40E-24 |
| COL6A1 | 3.34E-13 | 0.671865 | 0.432 | 0.348 | 7.41E-09 |
| NOTUM | 2.95E-13 | 0.667101 | 0.198 | 0.116 | 6.54E-09 |
| CRABP2 | 3.17E-24 | 0.665274 | 0.174 | 0.07 | 7.03E-20 |
| TWISTNB | 2.47E-14 | 0.664275 | 0.697 | 0.697 | 5.48E-10 |
| OGN | 3.36E-32 | 0.663175 | 0.393 | 0.237 | 7.45E-28 |
| FXYD1 | 1.09E-50 | 0.663043 | 0.775 | 0.691 | 2.41E-46 |
| PARM1 | 2.92E-26 | 0.652733 | 0.649 | 0.572 | 6.47E-22 |
| IGFBP4 | 1.31E-11 | 0.64799 | 0.79 | 0.878 | 2.90E-07 |
| IGFBP2 | 2.75E-26 | 0.643782 | 0.768 | 0.757 | 6.10E-22 |
| WNT4 | 2.11E-18 | 0.636485 | 0.409 | 0.304 | 4.68E-14 |
| CYP26B1 | 1.89E-48 | 0.63562 | 0.249 | 0.082 | 4.20E-44 |
| LAMP1 | 8.50E-32 | 0.634004 | 0.462 | 0.313 | 1.88E-27 |
| VAMP8 | 3.27E-20 | 0.628365 | 0.302 | 0.191 | 7.25E-16 |
| FBN2 | 5.84E-34 | 0.624472 | 0.38 | 0.211 | 1.29E-29 |
| SLC25A4 | 6.00E-40 | 0.624015 | 0.547 | 0.365 | 1.33E-35 |
| LBH | 1.64E-57 | 0.621446 | 0.255 | 0.071 | 3.64E-53 |
| FBLN5 | 5.70E-36 | 0.618509 | 0.748 | 0.648 | 1.26E-31 |
| SERINC1 | 5.81E-46 | 0.61593 | 0.625 | 0.454 | 1.29E-41 |
| FBLN1 | 5.21E-48 | 0.612491 | 0.794 | 0.688 | 1.16E-43 |
| CD74 | 8.58E-14 | 0.61232 | 0.186 | 0.103 | 1.90E-09 |
| ALDH1A2 | 9.49E-27 | 0.60386 | 0.517 | 0.393 | 2.10E-22 |
| COX16 | 6.20E-35 | 0.60385 | 0.706 | 0.632 | 1.37E-30 |
| GSTA1 | 1.14E-34 | 0.595045 | 0.201 | 0.067 | 2.52E-30 |
| PGRMC2 | 1.59E-31 | 0.594757 | 0.461 | 0.304 | 3.53E-27 |
| VAMP2 | 4.78E-38 | 0.588629 | 0.713 | 0.655 | 1.06E-33 |
| CD248 | 8.25E-25 | 0.585625 | 0.776 | 0.778 | 1.83E-20 |
| NPC2 | 1.82E-61 | 0.583092 | 0.96 | 0.933 | 4.04E-57 |
| LSP1 | 8.32E-26 | 0.575824 | 0.251 | 0.127 | 1.84E-21 |
| NFE2L1 | 1.79E-26 | 0.568322 | 0.544 | 0.43 | 3.96E-22 |
| QSOX1 | 2.03E-19 | 0.56532 | 0.445 | 0.334 | 4.51E-15 |
| TNFRSF21 | 9.14E-24 | 0.559502 | 0.404 | 0.274 | 2.03E-19 |
| MALAT1 | 2.61E-95 | 0.557937 | 0.995 | 0.995 | 5.79E-91 |

**Table S9: Top significant DE genes comparing fetal and maternal macrophages in placenta tissue, as identified by Originator.** Log2 fold change (avg_log2FC) is the averaged expression in the fetal compared to maternal macrophages.

|  | **p_val** | **avg_log2FC** | **pct.1** | **pct.2** | **p_val_adj** |
| --- | --- | --- | --- | --- | --- |
| SEPP1 | 7.59E-10 | 2.939086 | 0.863 | 0.296 | 1.68E-05 |
| ANKRD22 | 1.82E-07 | -0.31847 | 0.005 | 0.185 | 0.004043 |
| HLA-DOA | 5.87E-09 | -0.41583 | 0.005 | 0.222 | 0.00013 |
| FBXO6 | 3.80E-08 | -0.50193 | 0.016 | 0.259 | 0.000842 |
| CD38 | 6.69E-09 | -0.51085 | 0.005 | 0.222 | 0.000148 |
| SIGLEC10 | 1.39E-10 | -0.51302 | 0.005 | 0.259 | 3.08E-06 |
| FGR | 1.85E-09 | -0.55798 | 0.016 | 0.296 | 4.11E-05 |
| TAGAP | 3.68E-09 | -0.55826 | 0.027 | 0.333 | 8.17E-05 |
| SLAMF7 | 2.07E-07 | -0.57248 | 0.005 | 0.185 | 0.004591 |
| APOBEC3A | 1.76E-07 | -0.63216 | 0 | 0.148 | 0.003903 |
| PILRA | 1.11E-07 | -0.68662 | 0.066 | 0.407 | 0.002469 |
| AC147651.4 | 5.15E-09 | -0.71519 | 0.005 | 0.222 | 0.000114 |
| CLEC4E | 1.27E-10 | -0.73408 | 0 | 0.222 | 2.82E-06 |
| SSPN | 9.46E-08 | -0.7427 | 0.038 | 0.333 | 0.002096 |
| FCN1 | 5.17E-09 | -0.78535 | 0.011 | 0.259 | 0.000115 |
| ENO1 | 2.82E-07 | -0.91871 | 0.835 | 0.963 | 0.00624 |
| GBP1 | 2.32E-08 | -1.01877 | 0.088 | 0.481 | 0.000515 |
| FBP1 | 8.28E-09 | -1.04538 | 0.066 | 0.444 | 0.000184 |
| GBP2 | 9.71E-10 | -1.06374 | 0.066 | 0.481 | 2.15E-05 |
| CD48 | 4.85E-11 | -1.07269 | 0.066 | 0.519 | 1.08E-06 |
| HLA-DQA2 | 7.92E-14 | -1.09034 | 0.016 | 0.407 | 1.76E-09 |
| TYMP | 8.40E-10 | -1.10807 | 0.044 | 0.407 | 1.86E-05 |
| STAT1 | 7.60E-10 | -1.15925 | 0.071 | 0.481 | 1.69E-05 |
| TNFSF13B | 7.24E-08 | -1.27052 | 0.154 | 0.593 | 0.001605 |
| GCHFR | 2.23E-08 | -1.27756 | 0.242 | 0.778 | 0.000494 |
| HLA-A | 1.32E-07 | -1.29365 | 0.775 | 1 | 0.002918 |
| HLA-DMA | 2.39E-07 | -1.2983 | 0.302 | 0.741 | 0.005308 |
| FCGR3A | 3.41E-07 | -1.36724 | 0.385 | 0.852 | 0.007564 |
| LSP1 | 1.88E-10 | -1.38666 | 0.148 | 0.704 | 4.16E-06 |
| CXCL9 | 3.32E-12 | -1.4032 | 0 | 0.259 | 7.35E-08 |
| LAPTM5 | 7.70E-08 | -1.44247 | 0.522 | 0.963 | 0.001706 |
| CAPG | 3.11E-08 | -1.46339 | 0.324 | 0.852 | 0.00069 |
| HAMP | 6.41E-10 | -1.5656 | 0.033 | 0.37 | 1.42E-05 |
| SOD2 | 3.87E-07 | -1.66751 | 0.346 | 0.778 | 0.008586 |
| PLAUR | 4.33E-08 | -1.6812 | 0.225 | 0.704 | 0.00096 |
| TREM2 | 7.34E-11 | -1.8716 | 0.176 | 0.704 | 1.63E-06 |
| CXCR4 | 6.03E-13 | -1.99228 | 0.066 | 0.556 | 1.34E-08 |
| HLA-DRB1 | 7.34E-12 | -2.13116 | 0.39 | 0.963 | 1.63E-07 |
| CD52 | 4.03E-10 | -2.16276 | 0.082 | 0.519 | 8.93E-06 |
| APOC1 | 1.80E-10 | -2.28506 | 0.258 | 0.815 | 4.00E-06 |
| HLA-DQB1 | 3.08E-15 | -2.28714 | 0.154 | 0.815 | 6.82E-11 |
| CXCL10 | 2.56E-09 | -2.34346 | 0.011 | 0.259 | 5.66E-05 |
| PLIN2 | 4.16E-10 | -2.37356 | 0.363 | 0.889 | 9.22E-06 |
| CD74 | 3.73E-11 | -2.48105 | 0.632 | 1 | 8.26E-07 |
| HLA-DRB5 | 1.19E-07 | -2.52589 | 0.198 | 0.593 | 0.002643 |
| LYZ | 2.09E-11 | -2.59556 | 0.264 | 0.815 | 4.64E-07 |
| HLA-DPB1 | 1.63E-13 | -2.61009 | 0.247 | 0.889 | 3.61E-09 |
| RGS1 | 4.57E-18 | -2.61249 | 0.077 | 0.704 | 1.01E-13 |
| HLA-DQA1 | 1.03E-12 | -2.68723 | 0.11 | 0.63 | 2.27E-08 |
| HLA-DRA | 1.38E-12 | -2.86693 | 0.462 | 0.963 | 3.06E-08 |
| HLA-DPA1 | 9.05E-16 | -2.89269 | 0.28 | 0.963 | 2.01E-11 |

**Table S10: Cell-type-specific marker genes for placenta tissues**

| **cell type** | **genes** |
| --- | --- |
| Villous cytotrophoblast | PAGE4, PEG10, ISYNA1 |
| Syncytiotrophoblast | CGA, CYP19A1 |
| Extravillous trophoblast | HLA-G, HTRA4 |
| Fibroblast type 1 | DLK1, EGFL6, GPC3 |
| Fibroblast type 2 | DKK1, IGFBP5, IGFBP1 |
| Vascular endothelial cell | CD34, VWF, CLEC14A, ECSCR |
| T-cell | IL7R, CCR7 |
| NK cell | GNLY, NKG7 |
| Macrophage (HB) | LYVE1, DAB2, CCL4, CSF1R, CD163, CD209 |
| Monocyte | FCGR3A, MS4A7, CD14, LYZ |
| Erythrocyte | HBB, HBG1, HBA1 |

**Table S11: Cell-type-specific marker genes for PDAC tissues**

| **cell type** | **genes** |
| --- | --- |
| Epithelial cell | KRT19 |
| Ductal cell | SLC3A1, VTCN1, DCDC2, SERPINA5 |
| Acinar cell | PRSS3, PNLIP, CTRC |
| Endothelial cell | CD34, VWF, KDR |
| Peri-islet Schwann cell | GFRA3, MPZ, GFRA1, INSC, SOX10, S100B |
| Beta cell | INS, INS2, MAFA |
| Fibroblast | COL1A1, ACTA2 |
| M2 macrophage | CD163, PPARG, MRC1 |
| Myeloid-derived suppressor cell (MDSC) | S100A9, ICAM1, S100A8, CXCR1 |
| T-cell | IL7R, CCR7 |
| Regulatory T-cell | IKZF2, FOXP3, CTLA4 |
| NK cell | GNLY, NKG7, NCR1 |
| B-cell | MS4A1, CD19, BLK |
| Mast cell | KIT |
| Plasma cell | MZB1, SPAG4 |

**Table S12: Biological interpretation of common genes between 1) T-cell and T-reg, 2) macrophage and NK cell, and 3) NK cell, T-cell, and macrophage.**

| **Common cell types** | **Cell type** | **Gene** | **Highly express in** | | **Supporting citation** |
| --- | --- | --- | --- | --- | --- |
|  |  |  | **blood** | **Expected tissue-resident** |  |
| T- cell & T-reg | T-cell | MT-ND1 |  | ✓ | Lin et al. [1]; Rivadeinera & Delgoffe [2] |
|  |  | TNFRSF4 | ✓ |  | Ma et al. [3]; Iriki et al. [4]; Luo et al. [5] |
|  |  | RPS26 | ✓ |  | Chen et al. [6] |
|  |  | LTB | ✓ |  | Abdulrahman et al. [7] |
|  | T-reg | MT-ND1 |  | ✓ | Field et al. [8] |
|  |  | TNFRSF4 | ✓ |  | Chen et al. [9] |
|  |  | RPS26 | ✓ |  | Chen et al. [6] |
|  |  | LTB | ✓ |  | Abdulrahman et al. [7] |
| Macrophage & NK cell | Macrophage | RGCC |  | ✓ | Kamata & Tada [10]; Xu et al. [11] |
|  |  | CD63 | ✓ |  | Ye et al. [12]; Zhong et al. [13]; Khushman et al. [14] |
|  |  | LGALS1 | ✓ |  | Min et al. [15]; Murphy et al [16]; Schmieder & Schledzewski [17] |
|  | NK cell | RGCC |  | ✓ | Yu et al. [18] |
|  |  | CD63 | ✓ |  | Jewett et al. [19]; Khushman et al. [14] |
|  |  | LGALS1 | ✓ |  | Baker et al. [20]; Yu et al. [21] |
| NK cell, T-cell, & macrophage | NK cell | ZNF331 |  | ✓ | Marquardt et al. [22]; Foroutan et al. [23] |
|  | T-cell | ZNF331 | ✓ |  | Egelston et al. [24] |
|  | macrophage | ZNF331 | ✓ |  | Xiao et al. [25] |

**Reference**

1. Lin X, Zhou Y, Xue L. Mitochondrial complex I subunit MT-ND1 mutations affect disease progression. Heliyon. 2024 Apr;10(7):e28808.

2. Rivadeneira DB, Delgoffe GM. Antitumor T-cell Reconditioning: Improving Metabolic Fitness for Optimal Cancer Immunotherapy. Clin Cancer Res. 2018 Jun 1;24(11):2473–81.

3. Ma H, Feng P hui, Yu S ni, Lu Z hui, Yu Q, Chen J. Identification and validation of TNFRSF4 as a high-profile biomarker for prognosis and immunomodulation in endometrial carcinoma. BMC Cancer. 2022 Dec;22(1):543.

4. Iriki H, Takahashi H, Amagai M. Diverse Role of OX40 on T Cells as a Therapeutic Target for Skin Diseases. J Invest Dermatol. 2023 Apr;143(4):545–53.

5. Luo T, Wu Y, Chen Z, Tai Y, Zhang F, Chen W, et al. Multi-omics Analysis of Tumor Necrosis Factor Superfamily 4 Reveals Its Prognostic Value and T Cell Exhaustion Feature in Cancer [Internet]. 2023 [cited 2024 Dec 23]. Available from: https://www.researchsquare.com/article/rs-3174654/v1

6. Chen C, Peng J, Ma S, Ding Y, Huang T, Zhao S, et al. Ribosomal protein S26 serves as a checkpoint of T-cell survival and homeostasis in a p53-dependent manner. Cell Mol Immunol. 2021 Jul;18(7):1844–6.

7. Abdulrahman Z, Santegoets SJ, Sturm G, Charoentong P, Ijsselsteijn ME, Somarakis A, et al. Tumor-specific T cells support chemokine-driven spatial organization of intratumoral immune microaggregates needed for long survival. J Immunother Cancer. 2022 Feb;10(2):e004346.

8. Field CS, Baixauli F, Kyle RL, Puleston DJ, Cameron AM, Sanin DE, et al. Mitochondrial Integrity Regulated by Lipid Metabolism Is a Cell-Intrinsic Checkpoint for Treg Suppressive Function. Cell Metab. 2020 Feb;31(2):422-437.e5.

9. Chen X, Ma H, Mo S, Zhang Y, Lu Z, Yu S, et al. Analysis of the OX40/OX40L immunoregulatory axis combined with alternative immune checkpoint molecules in pancreatic ductal adenocarcinoma. Front Immunol. 2022 Jul 22;13:942154.

10. Kamata M, Tada Y. Dendritic Cells and Macrophages in the Pathogenesis of Psoriasis. Front Immunol. 2022 Jun 28;13:941071.

11. Xu L, Chen Y, Liu L, Hu X, He C, Zhou Y, et al. Tumor-associated macrophage subtypes on cancer immunity along with prognostic analysis and SPP1-mediated interactions between tumor cells and macrophages. Tang Y, editor. PLOS Genet. 2024 Apr 22;20(4):e1011235.

12. Ye H, Zhou Q, Zheng S, Li G, Lin Q, Wei L, et al. Tumor-associated macrophages promote progression and the Warburg effect via CCL18/NF-kB/VCAM-1 pathway in pancreatic ductal adenocarcinoma. Cell Death Dis. 2018 Apr 18;9(5):453.

13. Zhong W, Lu Y, Han X, Yang J, Qin Z, Zhang W, et al. Upregulation of exosome secretion from tumor-associated macrophages plays a key role in the suppression of anti-tumor immunity. Cell Rep. 2023 Oct;42(10):113224.

14. Khushman M, Patel GK, Laurini JA, Bhardwaj A, Roveda K, Donnell R, et al. Exosomal markers (CD63 and CD9) expression and their prognostic significance using immunohistochemistry in patients with pancreatic ductal adenocarcinoma. J Gastrointest Oncol. 2019 Aug;10(4):695–702.

15. Min Y, Huang R, Zhang H, Yang Q, Zhang Q, Chen D. 884P Prognostic value and immune characteristics of LGALS1 in head and neck squamous cell carcinoma. Ann Oncol. 2023 Oct;34:S567.

16. F Murphy J. Modulation of Angiogenesis by Tumor Associated Macrophages in the Tumor Microenvironment. MOJ Immunol [Internet]. 2014 Jul 24 [cited 2024 Dec 23];1(3). Available from: https://medcraveonline.com/MOJI/modulation-of-angiogenesis-by-tumor-associated-macrophages-in-the-tumor-microenvironment.html

17. Schmieder A, Schledzewski K. The Role of Tumor-Associated Macrophages (TAMs) in Tumor Progression. In: Klink M, editor. Interaction of Immune and Cancer Cells [Internet]. Vienna: Springer Vienna; 2014. p. 49–74. Available from: https://doi.org/10.1007/978-3-7091-1300-4_3

18. Yu Q, Shi X, Wang H, Zhang S, Hu S, Cai T. A Novel Prognostic Signature of comprising Nine NK Cell signatures Based on Both Bulk RNA Sequencing and Single-Cell RNA Sequencing for Hepatocellular Carcinoma. J Cancer. 2023;14(12):2209–23.

19. Jewett A, Kos J, Kaur K, Safaei T, Sutanto C, Chen W, et al. Natural Killer Cells: Diverse Functions in Tumor Immunity and Defects in Pre-neoplastic and Neoplastic Stages of Tumorigenesis. Mol Ther - Oncolytics. 2020 Mar;16:41–52.

20. Baker GJ, Chockley P, Yadav VN, Doherty R, Ritt M, Sivaramakrishnan S, et al. Natural Killer Cells Eradicate Galectin-1–Deficient Glioma in the Absence of Adaptive Immunity. Cancer Res. 2014 Sep 15;74(18):5079–90.

21. Yu X, Qian J, Ding L, Yin S, Zhou L, Zheng S. Galectin-1: A Traditionally Immunosuppressive Protein Displays Context-Dependent Capacities. Int J Mol Sci. 2023 Mar 30;24(7):6501.

22. Marquardt N, Kekäläinen E, Chen P, Lourda M, Wilson JN, Scharenberg M, et al. Unique transcriptional and protein-expression signature in human lung tissue-resident NK cells. Nat Commun. 2019 Aug 26;10(1):3841.

23. Foroutan M, Molania R, Pfefferle A, Behrenbruch C, Scheer S, Kallies A, et al. The Ratio of Exhausted to Resident Infiltrating Lymphocytes Is Prognostic for Colorectal Cancer Patient Outcome. Cancer Immunol Res. 2021 Oct 1;9(10):1125–40.

24. Egelston CA, Guo W, Tan J, Avalos C, Simons DL, Lim MH, et al. Tumor-infiltrating exhausted CD8+ T cells dictate reduced survival in premenopausal estrogen receptor–positive breast cancer. JCI Insight. 2022 Feb 8;7(3):e153963.

25. Xiao F, Shen J, Zhou L, Fang Z, Weng Y, Zhang C, et al. ZNF395 facilitates macrophage polarization and impacts the prognosis of glioma.
