# Supplementary material for: Single-cell RNA-seq data have prevalent blood contamination but can be rescued by Originator, a computational tool separating single-cell RNA-seq by genetic and contextual information
Source: Genome Biol. 2025 Mar 11;26:52. doi: 10.1186/s13059-025-03495-9 (PMC11895284; doi:10.1186/s13059-025-03495-9)

**Supplementary Notes**

**Testing the performance of Originator by an artificially mixed PBMC and breast cancer cell lines**

To evaluate the performance of the pipeline, we tested the tissue-blood deconvolution on artificially mixed tissue-blood resident data. This data were generated by combining an 8k PBMCs dataset from a healthy donor (blood-resident cells) and an *in vitro* cell mixture (expected tissue-resident cells) containing three breast cancer lines (T47D, BT474, MCF7), monocytes (Thp1), lymphocytes (Jukrat), and stem cells (hMSC) [[1,2](https://www.zotero.org/google-docs/?Q9U7t8)] **(Additional file 2: Fig. S1a-b)**. The result shows that the pipeline correctly separates 98.31% of cells (12,809 out of 13,029 cells) into blood and expected tissue-resident cells **(Additional file 2: Fig. S1c-d, Additional file 1: Table S2)**.

**Testing the performance of Originator by a paired clear cell renal cell carcinoma (ccRCC) tissue and PBMC from the same patients**

To evaluate the performance of Originator in separating blood and expected tissue-resident immune cells in a more realistic dataset with minimal batch effect, we used the dataset of Krishna et al. [[3](https://www.zotero.org/google-docs/?7rxe7M)] which contains paired ccRCC tissue and PBMC for the same patients. To retrieve the ground truth for ccRCC tissues without blood cells, we applied Originator on ccRCC tissues to remove potential blood cells to generate cleaned ccRCC tissues. These cleaned ccRCC tissues were subsequently combined with the paired PBMC to generate a mixture of blood and tissue-resident immune cells. We ran Originator on this artificial mixture of blood and tissue-resident immune cells for five iterations and calculated the F1 scores. Originator achieves F1 scores of 0.98, 0.93, 0.91, and 0.99 for B cell, CD4 T-cell, CD8 T-cell, and NK cell, respectively **(Additional file 2: Fig. S2)**.

**Benchmarking freemuxlet (in Originator) with scSplit, on separating single cells by genetic origins.**

We chose freemuxlet to separate single cells by genetic origins in Originator, as it does not require known references for SNPs in the samples. Its performance was benchmarked with another reference-free tool scSplit [[4](https://www.zotero.org/google-docs/?rb6vxD)] on two different datasets, one from our own placenta data, and another with the mixed PBMC data from two ccRCC patients [[3](https://www.zotero.org/google-docs/?Ddh6CT)]. The mixed PBMC dataset contains transcripts of 200 cells from each of two different individuals, with a total of 400 cells. Single Nucleotide Variants (SNV) were called with Freebayes [[5](https://www.zotero.org/google-docs/?gR7inz)] and fed into scSplit with the bam file. The results in both datasets indicate that freemuxlet outperforms scSplit in assigning cells back to their patient origin **(Additional file 1: Table S1)**.

**Other DE genes between blood and tissue for the PDAC dataset**

Besides significant DE genes in T-cells due to the microenvironment, macrophages have the most abundant DE genes between tumor and blood. For example, higher expression of INHBA is present in expected tissue-resident compared to blood macrophages **(Additional file 2: Fig. S5b)**, consistent with the previously reported upregulation of INHBA by M2-phenotype macrophages in pancreatic cancer **(Additional file 1: Table S5)** [[6](https://www.zotero.org/google-docs/?eeD1u0)]. We observed higher expression of GZMK in expected tissue-resident NK cells compared to those in blood, consistent with the previously reported expression of cytotoxicity signatures comparing blood and tumor NK cell populations **(Additional file 2: Figure S5c, Additional file 1: Table S4)** [[7](https://www.zotero.org/google-docs/?IuTc9o)]. We also observed higher expression of LILRA5 in expected tissue-resident monocytes than those from blood **(Additional file 2: Fig. S5d, Additional file 1: Table S6)**. This is consistent with the previous study, which found that the cross-linking of LILRA5 on monocytes induces the production of pro-inflammatory cytokines, suggesting the inflammatory response associated with PDAC [[8](https://www.zotero.org/google-docs/?1T1L53)].

Some DE genes between expected tissue-resident and blood immune cells are common among different immune cell types **(Additional file 2: Fig. S8)**. We observed that MT-ND1, TNFRSF4, RPS26, and LTB are differentially expressed in both T-cell and T-reg in PDAC tissues compared to those in blood. MT-ND1 has higher expression in the tissue compartment compared to the blood, consistent with the previous study [[9](https://www.zotero.org/google-docs/?WvYX3r)]. On the other hand, the expression of TNFRSF4, RPS26, and LTB is decreased in T-cell and T-reg in PDAC tissues compared to those in blood, with RPS26 showing higher expression levels than the other two genes. MT-ND1 is crucial for ATP production, and its high expression in tissue-resident T-cells and T-reg may reflect elevated energy demands necessary for effective immune responses against tumors [[10](https://www.zotero.org/google-docs/?5wZsgI)]. In T-reg, mtDNA, including MT-ND1, was shown to be increased in tumor tissue due to mitochondrial abnormalities to drive cGAS-STING signaling [[11](https://www.zotero.org/google-docs/?PeuT9A)]. RPS26 is a ribosomal protein-encoding gene and plays a key role in regulating their survival [[12](https://www.zotero.org/google-docs/?WAH5Pq)]. The decreased RPS26 expression in the T-cells and T-regs in the tumor tissue suggests the survival impairment of both cell types in PDAC.

We observed that RGCC, CD63, and LGALS1 are differentially expressed in both macrophages in NK cells. RGCC has higher expression in tissue-resident macrophages in PDAC tissues, consistent with the previous study that RGCC is highly expressed in M2 macrophages in tumor tissues [[13,14](https://www.zotero.org/google-docs/?N5NOEO)]. It is also highly expressed in tissue-resident NK cells compared in the blood, consistent with the previous study [[15](https://www.zotero.org/google-docs/?2CMuSO)]. Conversely, CD63 and LGALS1 are decreased in macrophages and NK cells in tissues compared to the blood. CD63 is an M2 macrophage marker in PDAC [[16](https://www.zotero.org/google-docs/?QP5whp)]. Reduced CD63 may impair exosome production and secretion in the macrophages, leading to diminished intercellular communication necessary for immune activation within the TME [[17,18](https://www.zotero.org/google-docs/?5s6Aeu)]. CD63 is expressed when NK cells are activated and ready to release cytotoxic granules; decreased expression of CD63 in NK cells may suggest impaired NK cell function within PDAC TME [[18,19](https://www.zotero.org/google-docs/?Ute5zr)]. LGALS1 was previously shown to regulate tumor infiltration of macrophages [[20](https://www.zotero.org/google-docs/?sRQKP7)]. Lower LGALS1 levels in tissue macrophages might reduce its infiltration, potentially impacting stromal remodeling [[21,22](https://www.zotero.org/google-docs/?XkuMhI)].

ZNF331 is differentially expressed in NK cells, T-cells, and macrophages in the dataset. In NK cells, ZNF331 is higher in tissue-resident NK cells compared to those in blood. This is consistent with the expression of ZNF331 in tissue-resident NK cells in previous studies [[23,24](https://www.zotero.org/google-docs/?bgqbhz)]. However, ZNF331 is decreased in T-cells and macrophages in tissues compared to those in blood. Upregulation of ZNF331 expression in tissue-resident T-cells was shown to dictate its cell cycle regulation [[25](https://www.zotero.org/google-docs/?A2yPuR)]. Decreased ZNF331 expression in tissue-resident T cells in PDAC tissue may impair the ability to proliferate and function effectively, and lead to compromised anti-tumor immunity. Though ZNF331 expression on macrophages is not extensively studied, Zinc finger protein was previously shown to play a role in macrophage polarization [[26](https://www.zotero.org/google-docs/?Ax7ECz)]. Deregulated ZNF331 in tissue-resident macrophages may impair the polarization of macrophages and the anti-tumor function.

**Reference:**

[1.](https://www.zotero.org/google-docs/?UqI2Zc) 10x Genomics. 8k PBMCs from a Healthy Donor, Single Cell Gene Expression Dataset by Cell Ranger 1.3.0. Datasets. 10x Genomics Datasets.<https://support.10xgenomics.com/single-cell-gene-expression/datasets/1.3.0/pbmc8k> (2017).

[2.](https://www.zotero.org/google-docs/?UqI2Zc) Sumazin P. GSE220606, Effective methods for bulk RNA-seq deconvolution using scnRNA-seq transcriptomes [cell mixtures scRNA-seq]. Datasets. Gene Expression Omnibus.<https://www.ncbi.nlm.nih.gov/geo/query/acc.cgi?acc=GSE220606> (2023).

[3. Krishna C, DiNatale RG, Kuo F, Srivastava RM, Vuong L, Chowell D, et al. Single-cell sequencing links multiregional immune landscapes and tissue-resident T cells in ccRCC to tumor topology and therapy efficacy. Cancer Cell [Internet]. 2021 May [cited 2024 May 31];39(5):662-677.e6. Available from: https://linkinghub.elsevier.com/retrieve/pii/S1535610821001653](https://www.zotero.org/google-docs/?UqI2Zc)

[4. Xu J, Falconer C, Nguyen Q, Crawford J, McKinnon BD, Mortlock S, et al. Genotype-free demultiplexing of pooled single-cell RNA-seq. Genome Biol [Internet]. 2019 Dec [cited 2024 Dec 23];20(1):290. Available from: https://genomebiology.biomedcentral.com/articles/10.1186/s13059-019-1852-7](https://www.zotero.org/google-docs/?UqI2Zc)

[5. Garrison E, Marth G. Haplotype-based variant detection from short-read sequencing [Internet]. arXiv; 2012 [cited 2024 Dec 24]. Available from: http://arxiv.org/abs/1207.3907](https://www.zotero.org/google-docs/?UqI2Zc)

[6. Liang Z, Yu J, Gu D, Liu X, Liu J, Wu M, et al. M2‐phenotype tumour‐associated macrophages upregulate the expression of prognostic predictors MMP14 and INHBA in pancreatic cancer. J Cell Mol Med [Internet]. 2022 Mar [cited 2024 Feb 27];26(5):1540–55. Available from: https://onlinelibrary.wiley.com/doi/10.1111/jcmm.17191](https://www.zotero.org/google-docs/?UqI2Zc)

[7. De Andrade LF, Lu Y, Luoma A, Ito Y, Pan D, Pyrdol JW, et al. Discovery of specialized NK cell populations infiltrating human melanoma metastases. JCI Insight [Internet]. 2019 Dec 5 [cited 2024 Feb 27];4(23):e133103. Available from: https://insight.jci.org/articles/view/133103](https://www.zotero.org/google-docs/?UqI2Zc)

[8. Mitchell A, Rentero C, Endoh Y, Hsu K, Gaus K, Geczy C, et al. LILRA5 is expressed by synovial tissue macrophages in rheumatoid arthritis, selectively induces pro‐inflammatory cytokines and IL‐10 and is regulated by TNF‐α, IL‐10 and IFN‐γ. Eur J Immunol [Internet]. 2008 Dec [cited 2024 Mar 25];38(12):3459–73. Available from: https://onlinelibrary.wiley.com/doi/10.1002/eji.200838415](https://www.zotero.org/google-docs/?UqI2Zc)

[9. Lin X, Zhou Y, Xue L. Mitochondrial complex I subunit MT-ND1 mutations affect disease progression. Heliyon [Internet]. 2024 Apr [cited 2024 Dec 23];10(7):e28808. Available from: https://linkinghub.elsevier.com/retrieve/pii/S2405844024048394](https://www.zotero.org/google-docs/?UqI2Zc)

[10. Rivadeneira DB, Delgoffe GM. Antitumor T-cell Reconditioning: Improving Metabolic Fitness for Optimal Cancer Immunotherapy. Clin Cancer Res [Internet]. 2018 Jun 1 [cited 2024 Dec 23];24(11):2473–81. Available from: https://aacrjournals.org/clincancerres/article/24/11/2473/80785/Antitumor-T-cell-Reconditioning-Improving](https://www.zotero.org/google-docs/?UqI2Zc)

[11. Field CS, Baixauli F, Kyle RL, Puleston DJ, Cameron AM, Sanin DE, et al. Mitochondrial Integrity Regulated by Lipid Metabolism Is a Cell-Intrinsic Checkpoint for Treg Suppressive Function. Cell Metab [Internet]. 2020 Feb [cited 2024 Dec 23];31(2):422-437.e5. Available from: https://linkinghub.elsevier.com/retrieve/pii/S1550413119306655](https://www.zotero.org/google-docs/?UqI2Zc)

[12. Chen C, Peng J, Ma S, Ding Y, Huang T, Zhao S, et al. Ribosomal protein S26 serves as a checkpoint of T-cell survival and homeostasis in a p53-dependent manner. Cell Mol Immunol [Internet]. 2021 Jul [cited 2024 Dec 23];18(7):1844–6. Available from: https://www.nature.com/articles/s41423-021-00699-4](https://www.zotero.org/google-docs/?UqI2Zc)

[13. Kamata M, Tada Y. Dendritic Cells and Macrophages in the Pathogenesis of Psoriasis. Front Immunol [Internet]. 2022 Jun 28 [cited 2024 Dec 23];13:941071. Available from: https://www.frontiersin.org/articles/10.3389/fimmu.2022.941071/full](https://www.zotero.org/google-docs/?UqI2Zc)

[14. Xu L, Chen Y, Liu L, Hu X, He C, Zhou Y, et al. Tumor-associated macrophage subtypes on cancer immunity along with prognostic analysis and SPP1-mediated interactions between tumor cells and macrophages. Tang Y, editor. PLOS Genet [Internet]. 2024 Apr 22 [cited 2024 Dec 23];20(4):e1011235. Available from: https://dx.plos.org/10.1371/journal.pgen.1011235](https://www.zotero.org/google-docs/?UqI2Zc)

[15. Yu Q, Shi X, Wang H, Zhang S, Hu S, Cai T. A Novel Prognostic Signature of comprising Nine NK Cell signatures Based on Both Bulk RNA Sequencing and Single-Cell RNA Sequencing for Hepatocellular Carcinoma. J Cancer [Internet]. 2023 [cited 2024 Dec 23];14(12):2209–23. Available from: https://www.jcancer.org/v14p2209.htm](https://www.zotero.org/google-docs/?UqI2Zc)

[16. Ye H, Zhou Q, Zheng S, Li G, Lin Q, Wei L, et al. Tumor-associated macrophages promote progression and the Warburg effect via CCL18/NF-kB/VCAM-1 pathway in pancreatic ductal adenocarcinoma. Cell Death Dis [Internet]. 2018 Apr 18 [cited 2024 Dec 23];9(5):453. Available from: https://www.nature.com/articles/s41419-018-0486-0](https://www.zotero.org/google-docs/?UqI2Zc)

[17. Zhong W, Lu Y, Han X, Yang J, Qin Z, Zhang W, et al. Upregulation of exosome secretion from tumor-associated macrophages plays a key role in the suppression of anti-tumor immunity. Cell Rep [Internet]. 2023 Oct [cited 2024 Dec 23];42(10):113224. Available from: https://linkinghub.elsevier.com/retrieve/pii/S2211124723012366](https://www.zotero.org/google-docs/?UqI2Zc)

[18. Khushman M, Patel GK, Laurini JA, Bhardwaj A, Roveda K, Donnell R, et al. Exosomal markers (CD63 and CD9) expression and their prognostic significance using immunohistochemistry in patients with pancreatic ductal adenocarcinoma. J Gastrointest Oncol [Internet]. 2019 Aug [cited 2024 Dec 23];10(4):695–702. Available from: http://jgo.amegroups.com/article/view/22798/21578](https://www.zotero.org/google-docs/?UqI2Zc)

[19. Jewett A, Kos J, Kaur K, Safaei T, Sutanto C, Chen W, et al. Natural Killer Cells: Diverse Functions in Tumor Immunity and Defects in Pre-neoplastic and Neoplastic Stages of Tumorigenesis. Mol Ther - Oncolytics [Internet]. 2020 Mar [cited 2024 Dec 23];16:41–52. Available from: https://linkinghub.elsevier.com/retrieve/pii/S2372770519301007](https://www.zotero.org/google-docs/?UqI2Zc)

[20. Min Y, Huang R, Zhang H, Yang Q, Zhang Q, Chen D. 884P Prognostic value and immune characteristics of LGALS1 in head and neck squamous cell carcinoma. Ann Oncol [Internet]. 2023 Oct [cited 2024 Dec 23];34:S567. Available from: https://linkinghub.elsevier.com/retrieve/pii/S0923753423028661](https://www.zotero.org/google-docs/?UqI2Zc)

[21. F Murphy J. Modulation of Angiogenesis by Tumor Associated Macrophages in the Tumor Microenvironment. MOJ Immunol [Internet]. 2014 Jul 24 [cited 2024 Dec 23];1(3). Available from: https://medcraveonline.com/MOJI/modulation-of-angiogenesis-by-tumor-associated-macrophages-in-the-tumor-microenvironment.html](https://www.zotero.org/google-docs/?UqI2Zc)

[22. Schmieder A, Schledzewski K. The Role of Tumor-Associated Macrophages (TAMs) in Tumor Progression. In: Klink M, editor. Interaction of Immune and Cancer Cells [Internet]. Vienna: Springer Vienna; 2014. p. 49–74. Available from: https://doi.org/10.1007/978-3-7091-1300-4_3](https://www.zotero.org/google-docs/?UqI2Zc)

[23. Marquardt N, Kekäläinen E, Chen P, Lourda M, Wilson JN, Scharenberg M, et al. Unique transcriptional and protein-expression signature in human lung tissue-resident NK cells. Nat Commun [Internet]. 2019 Aug 26 [cited 2024 Dec 23];10(1):3841. Available from: https://www.nature.com/articles/s41467-019-11632-9](https://www.zotero.org/google-docs/?UqI2Zc)

[24. Foroutan M, Molania R, Pfefferle A, Behrenbruch C, Scheer S, Kallies A, et al. The Ratio of Exhausted to Resident Infiltrating Lymphocytes Is Prognostic for Colorectal Cancer Patient Outcome. Cancer Immunol Res [Internet]. 2021 Oct 1 [cited 2024 Dec 23];9(10):1125–40. Available from: https://aacrjournals.org/cancerimmunolres/article/9/10/1125/665561/The-Ratio-of-Exhausted-to-Resident-Infiltrating](https://www.zotero.org/google-docs/?UqI2Zc)

[25. Egelston CA, Guo W, Tan J, Avalos C, Simons DL, Lim MH, et al. Tumor-infiltrating exhausted CD8+ T cells dictate reduced survival in premenopausal estrogen receptor–positive breast cancer. JCI Insight [Internet]. 2022 Feb 8 [cited 2024 Dec 23];7(3):e153963. Available from: https://insight.jci.org/articles/view/153963](https://www.zotero.org/google-docs/?UqI2Zc)

[26. Xiao F, Shen J, Zhou L, Fang Z, Weng Y, Zhang C, et al. ZNF395 facilitates macrophage polarization and impacts the prognosis of glioma.](https://www.zotero.org/google-docs/?UqI2Zc)

**Supplementary Figures**

**Fig. S1: Originator accurately separates artificially mixed blood cells from expected tissue-resident cells. a,** Data generation. **b,** UMAP of artificially mixed blood and tissue-resident data grouped by cell types. **c,** UMAP of artificially mixed blood-tissue resident data grouped by the data sources. **d,** UMAP of blood immune and expected tissue-resident cells recovered by Originator. **e-f,** UMAP of the alignment of whole blood reference, and artificially mixed blood-tissue resident data after batch correction using Harmony grouped by data sources (e) and cell types (f)

**
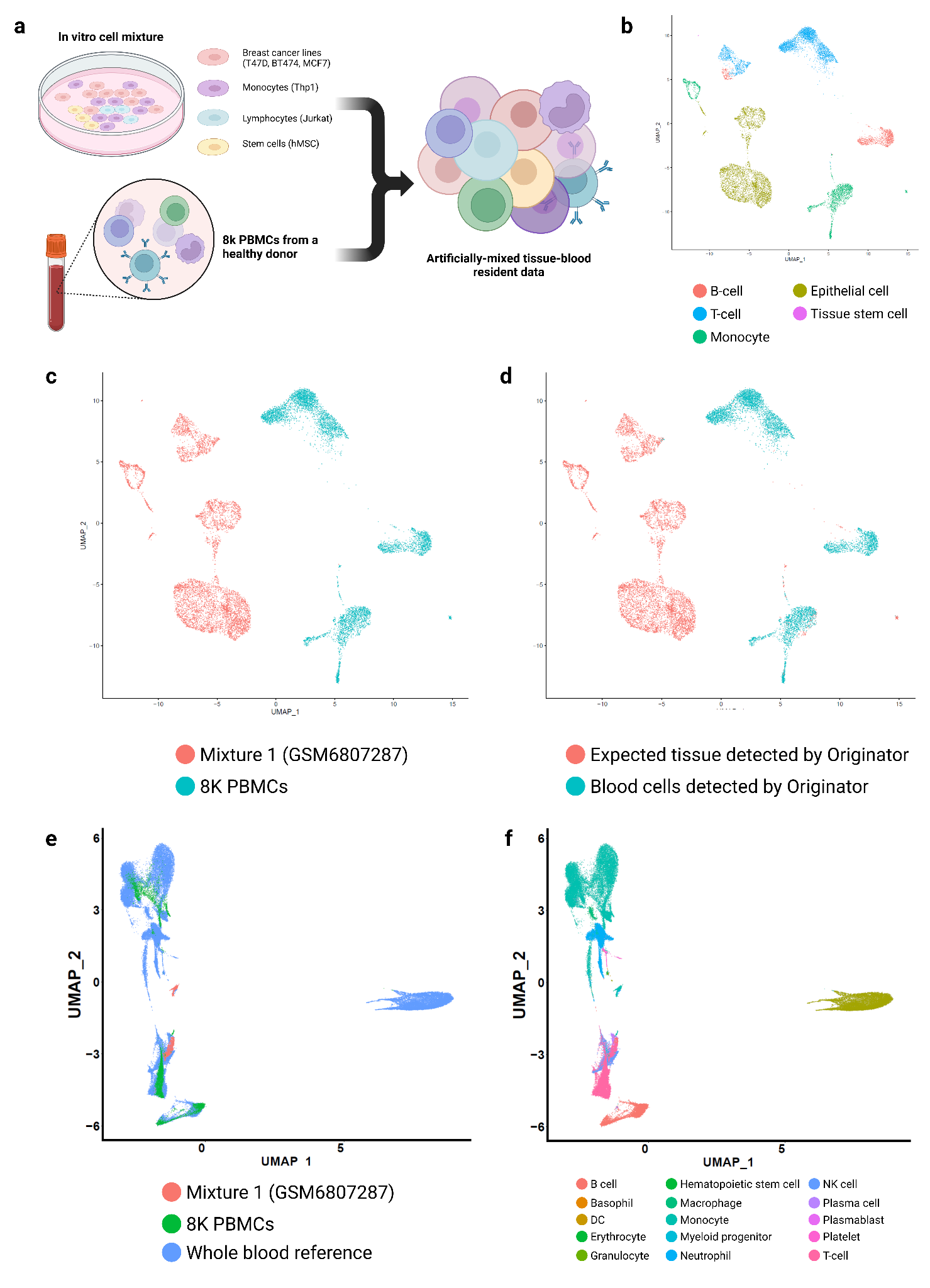
**

**Fig. S2: Originator accurately separates immune cells in the blood from those expected in the tissue of the same patient.** To generate the “ground truth” cell types for blood-eliminated ccRCC tissue, we applied Originator (or Seurat, for comparison) to ccRCC to remove the potential blood immune cells from ccRCC tissue first. We then integrated the cleaned ccRCC tissues and PBMC to generate an artificial mixture. Next we ran Originator on this pre-cleaned mixture dataset for five iterations.The boxplot shows the average F1 scores of separating immune cell types in the blood vs. blood-eliminated ccRCC tissue using Originator, where blood-elimination in the ccRCC data were done by Originator-based and Seurat-based approach, as stated above.

**
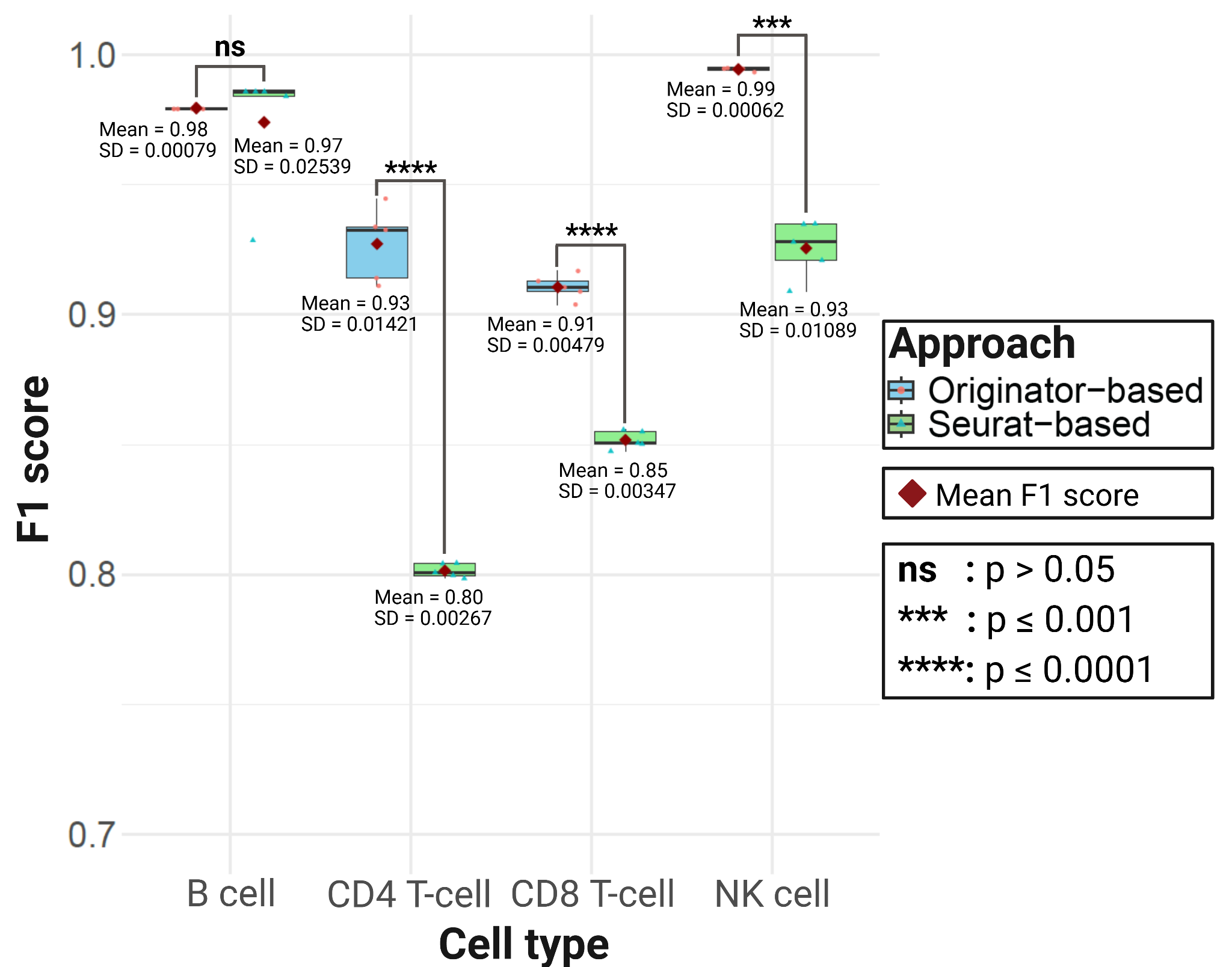
**

**Fig. S3:** **Blood and expected tissue-resident immune cells in healthy tissue datasets identified by Originator.** **a-b,** lung tissue datasets. **c,** spleen tissue datasets. **d-f,** liver tissue datasets.


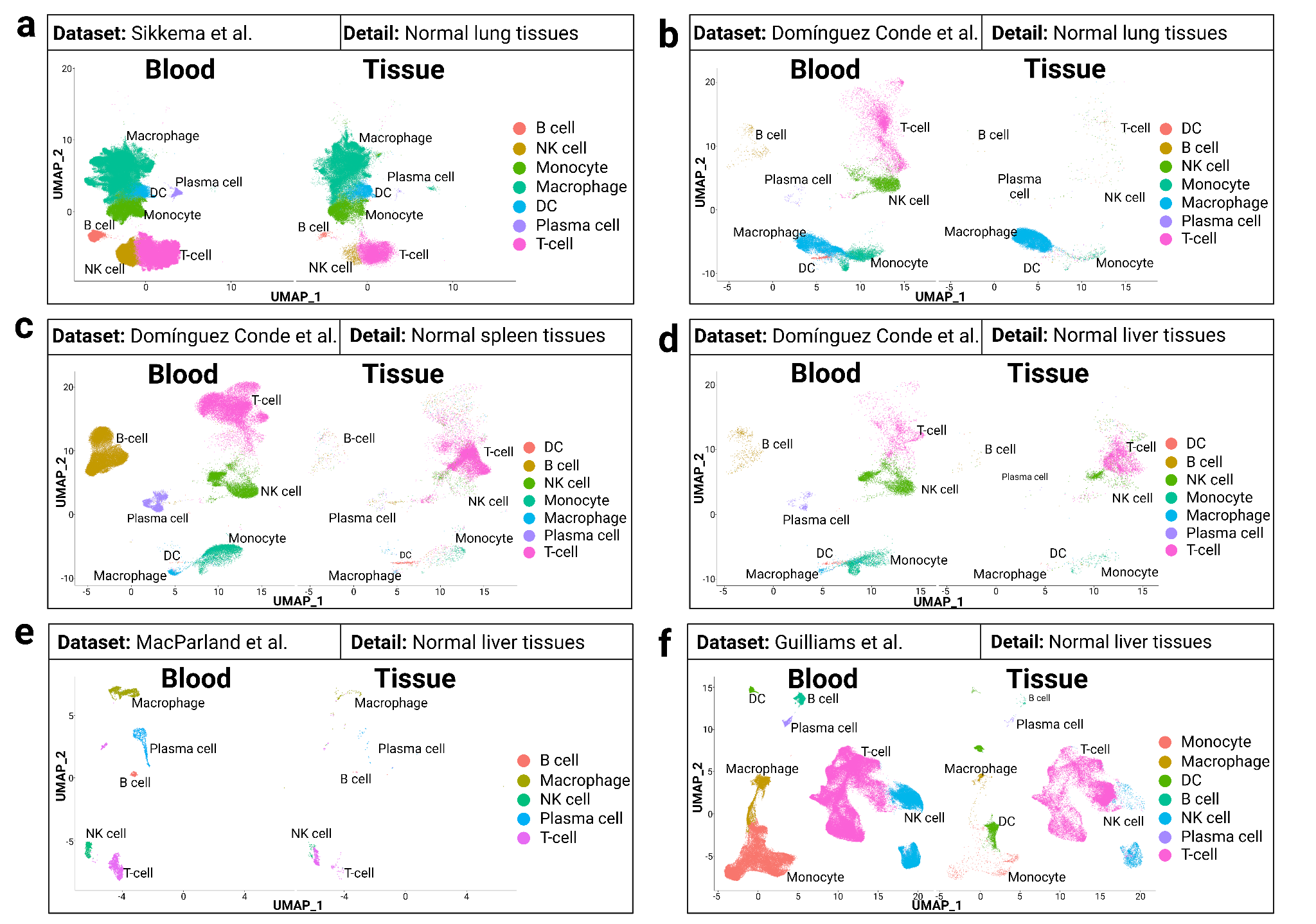


**Fig. S4:** **Blood and expected tissue-resident immune cells in cancer tissue datasets identified by Originator. a-b,** lung cancer tissue datasets. **C,** kidney cancer tissue dataset.


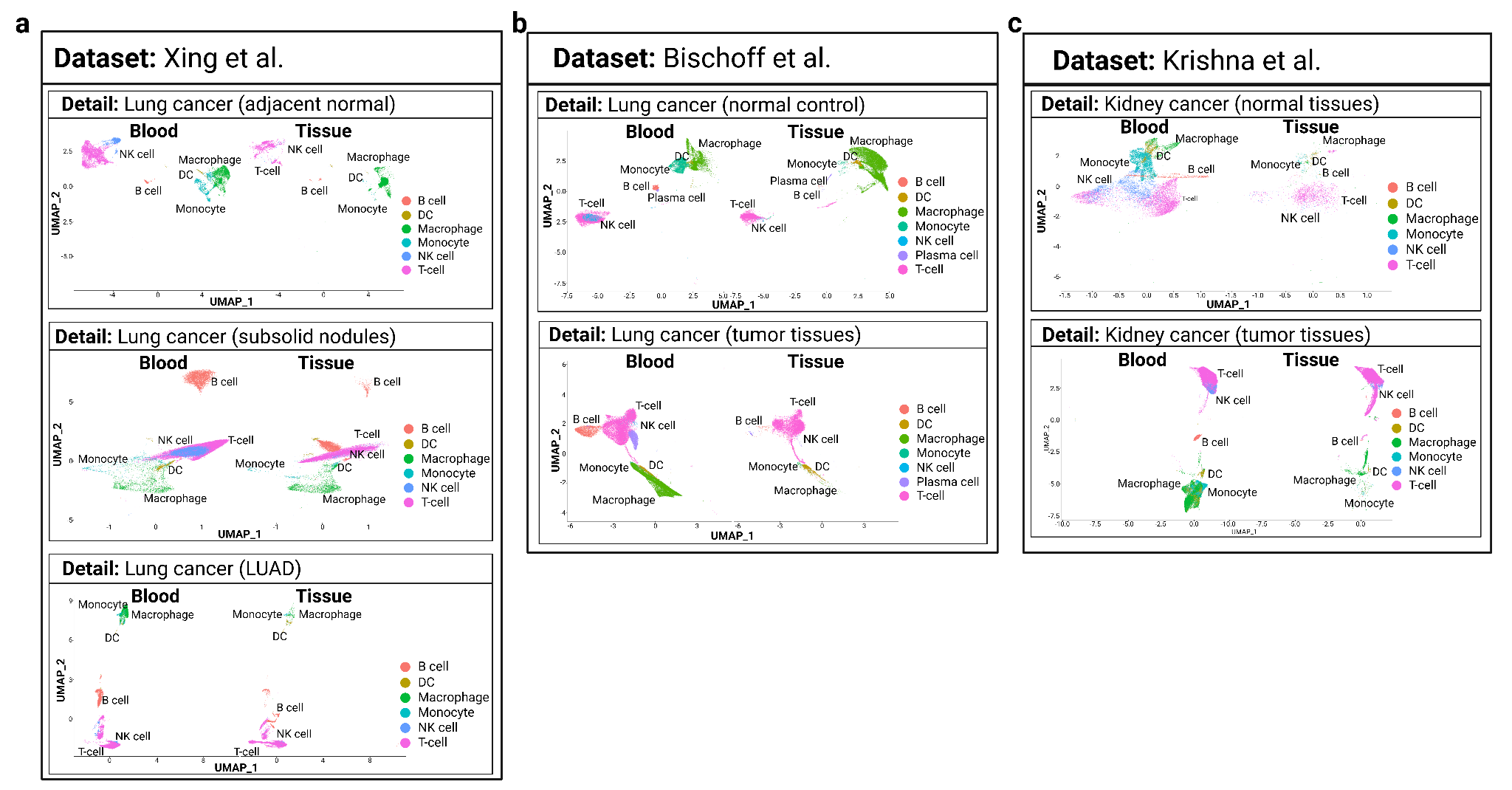


**Fig. S5: UMAP displaying the expression of DE genes in immune cells from blood vs. expected PDAC tumor-resident immune cells. a,** CCL4 and CCL5 expression in T-cells. **b,** INHBA expression in macrophages. **c,** GZMK expression in NK cells. **d),** LILRA5 expression in monocytes.


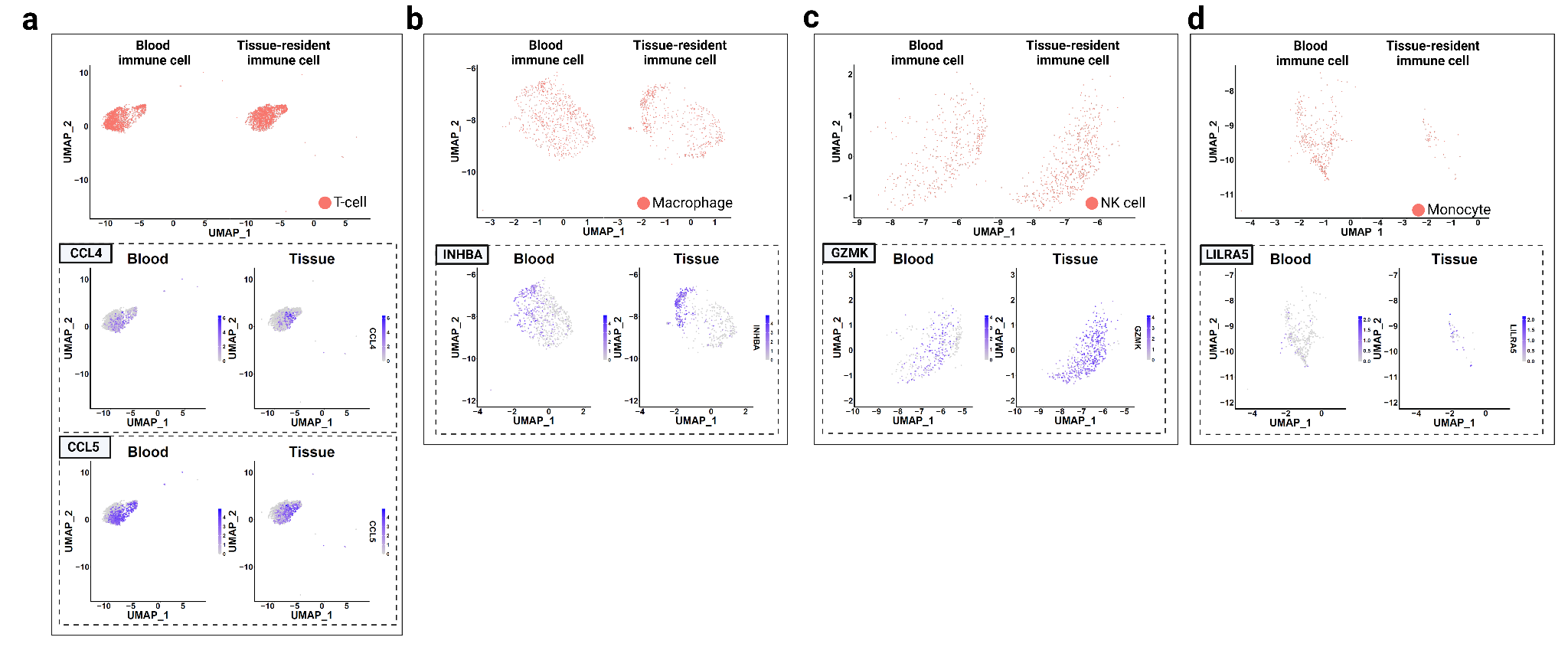


**Fig. S6: Expression of DE genes in common cell types between fetal and maternal tissues of placenta. a,** EGFL6 expression in fibroblast type 1 and 2. **b,** SEPP1 expression in macrophages (HB)


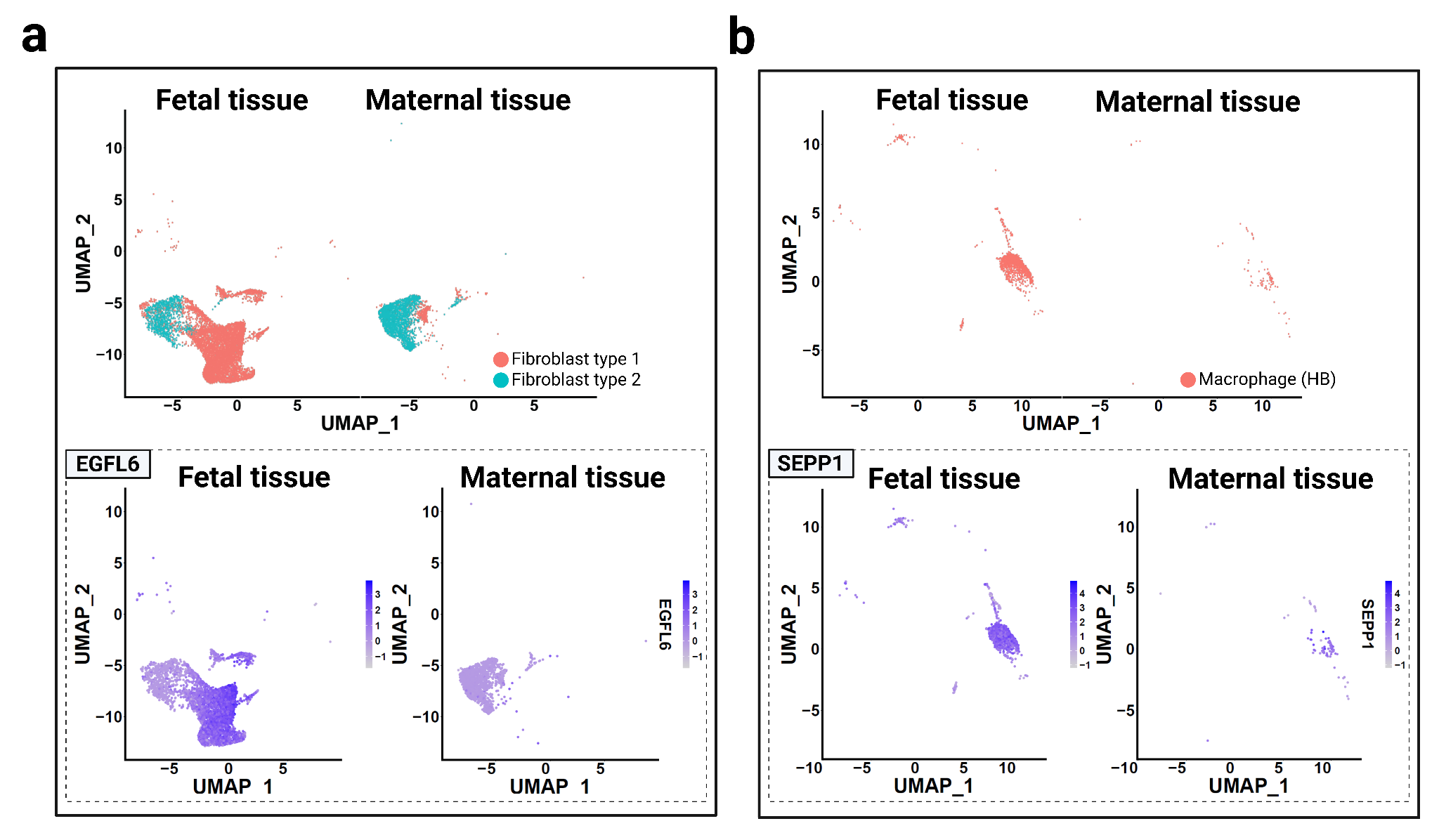


**Fig. S7: Comparison of PCA-based and UMAP-based Originator on assigning cells from paired ccRCC tissues and PBMC data provided by Krishna et al. 2021**


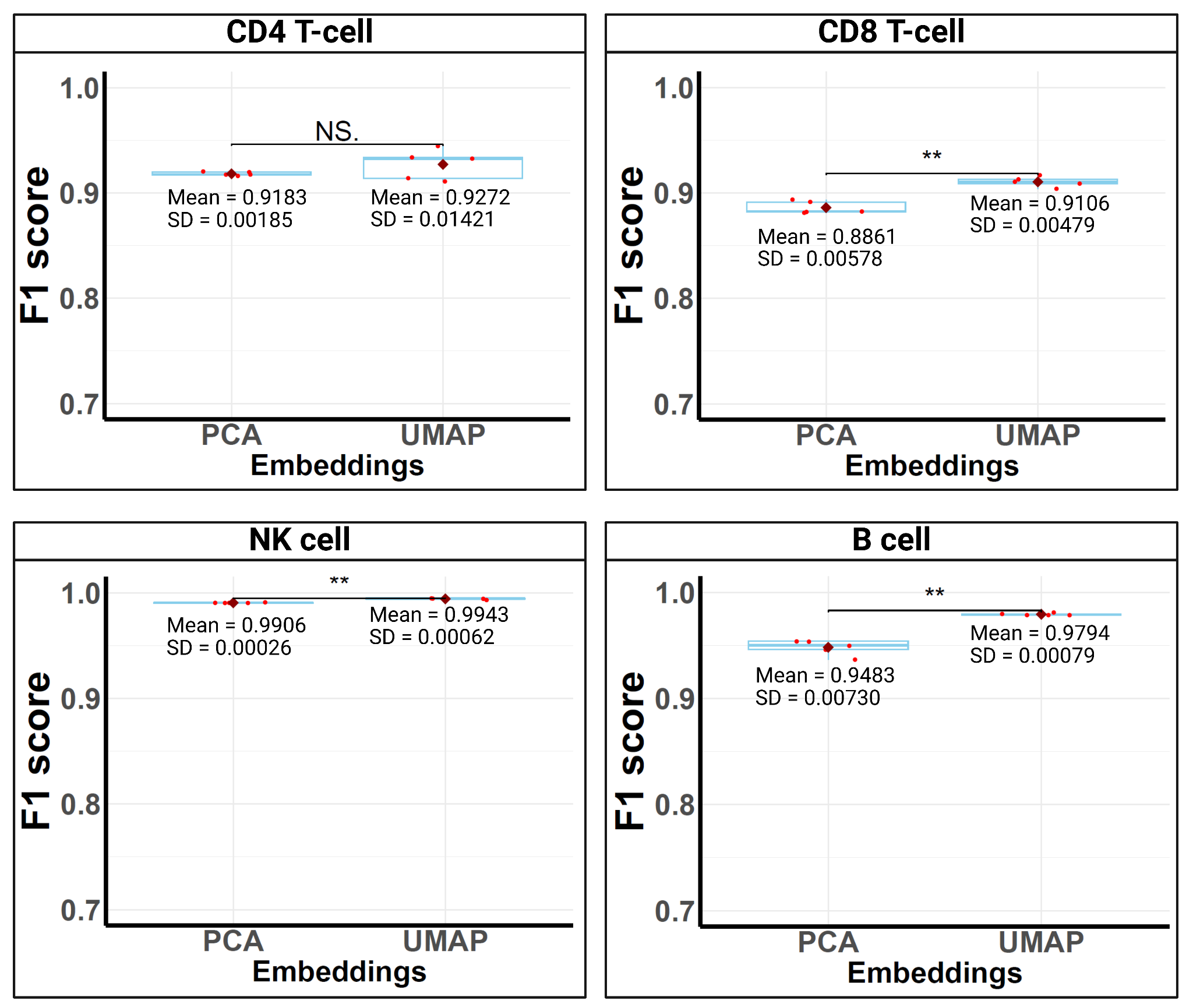


****: P ≤ 0.01**

**Fig. S8: UMAP plots showing the expression of DE genes between blood vs tissue among some common immune cell types;** A, UMAP of blood and tissue-resident immune cell., (b-e): DE genes common between T-cells and T-regs. (b) MT-ND1 (c) TNFRSF4 (d) RPS26 (e) LTB (f-h): DE genes common between macrophage and NK cells (f) RGCC (g) CD63. (h) LGALS1; (i): ZNF331,common in T-cell, NK cell, and macrophage cells


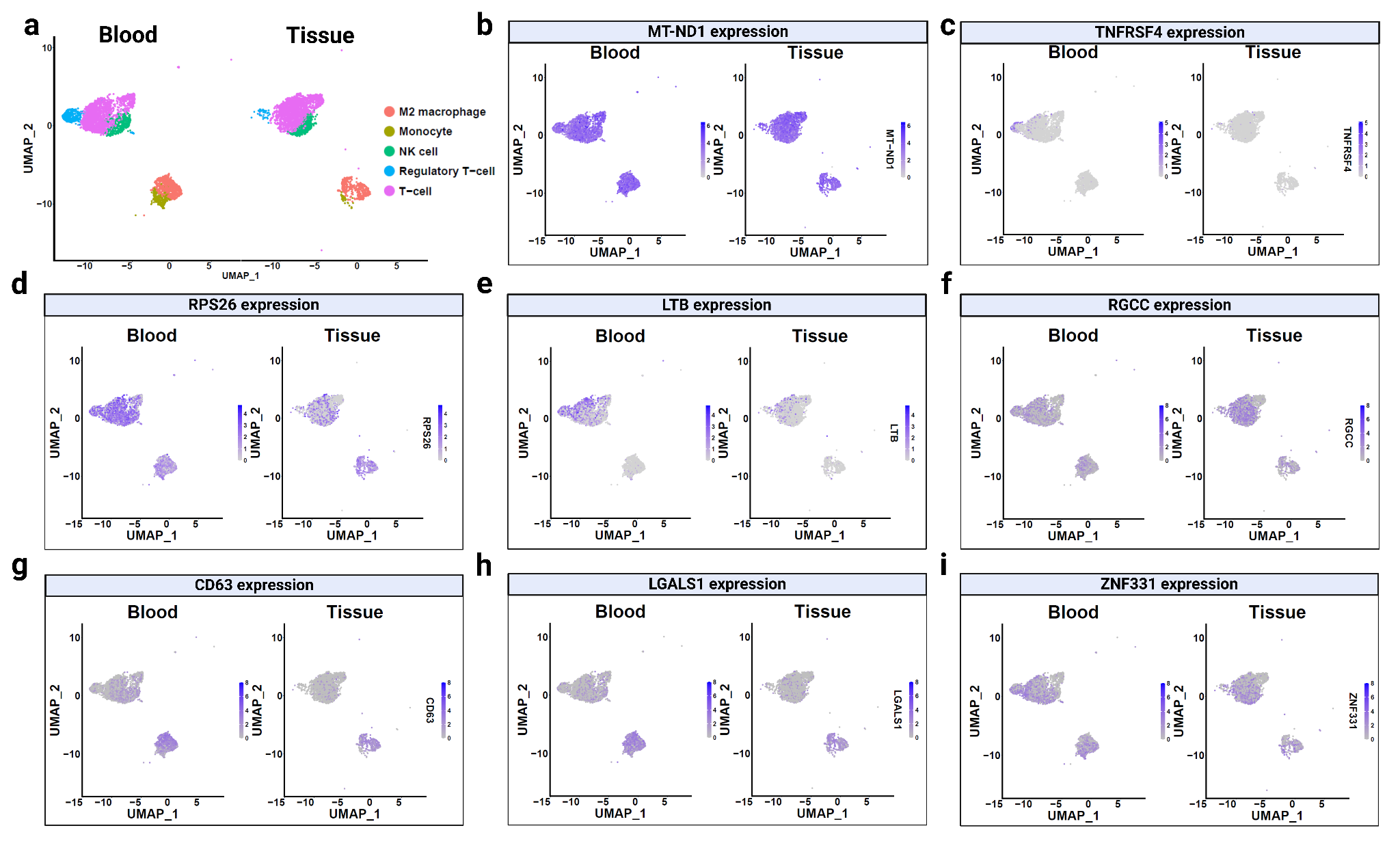

Supplement: Supplementary file 2 — Additional file 2: Supplementary Notes, Figs. S1-S8. [file 13059_2025_3495_MOESM2_ESM.docx]
